# Pan-cancer landscape of CENPO and its underlying mechanism in LUAD

**DOI:** 10.1186/s12931-023-02408-3

**Published:** 2023-04-15

**Authors:** Tongdong Shi, Zaoxiu Hu, Li Tian, Yanlong Yang

**Affiliations:** 1 Department of Thoracic Surgery, The First Affiliated Hospital of Kunming Medical University, No.295 Xichang Road, Wuhua District, Kunming, 650032 Yunnan People’s Republic of China; 2 Department of Infectious Diseases, Key Laboratory of Molecular Biology for Infectious Diseases and Institute for Viral Hepatitis, Chongqing Medical University the Second Affiliated Hospital, 74 Linjiang Road, Chongqing, 400010 People’s Republic of China; 3 Department of Pathology, The Third Affiliated Hospital of Kunming Medical University, No.519 Kunzhou Road, Xishan District, Kunming, 650118 Yunnan People’s Republic of China; 4 Department of Infectious Diseases, Key Laboratory of Molecular Biology for Infectious Diseases (Ministry of Education), The Second Affiliated Hospital of Chongqing Medical University, No.288 Tianwen Avenue, Nan’an District, Chongqing, 401336 People’s Republic of China

**Keywords:** Pan-cancer, CENPO, Immune infiltration, LUAD, Cell growth, mTOR signaling

## Abstract

**Background:**

Centromere protein O (CENPO) is a newly discovered constitutive centromeric protein, associated with cell death. However, little is known about how CENPO expression is associated with human cancers or immune infiltration. Here, we assessed the function of CENPO in pan-cancer and further verified the results in lung adenocarcinoma (LUAD) through in vitro and in vivo experiments.

**Methods:**

Sangerbox and TCGA databases were used to evaluate the CENPO expression level in different human cancer types. A subsequent evaluation of the potential role of CENPO as a diagnostic and prognostic biomarker in pancancer was conducted. The CENPO mutations were analyzed using the cBioPortal database and its function was analyzed using the LinkedOmics and CancerSEA databases. The TIMER2 and TISIDB websites were used to find out how CENPO affects immune infiltration. The expression level of CENPO in LUAD was revealed by TCGA database and immunohistochemical (IHC) staining. Targetscan, miRWalk, miRDB, miRabel, LncBase databases, and Cytoscape tool were used to identify microRNAs (miRNAs) and long noncoding RNAs (lncRNAs) that regulate expression and construct ceRNA network. Subsequently, loss-of-function assays were performed to identify the functions of CENPO on the malignant behavior and tumor growth of LUAD in vitro and in vivo experiments.

**Results:**

In most cancers, CENPO was upregulated and mutated, which predicted a poorer prognosis. Furthermore, infiltration of CENPO and myeloid-derived suppressor cells (MDSC) showed a significant positive correlation, while T-cell NK infiltration showed a significant negative correlation in most cancers. CENPO was expressed at high levels in LUAD and was correlated with p-TNM stage. Furthermore, CENPO knockdown suppressed the malignant phenotypes of LUAD cells, manifested by slower proliferation, cycle in G2, increased apoptosis, decreased migration, and attenuated tumorigenesis. Furthermore, CENPO knockdown decreased CDK1/6, PIK3CA, and inhibited mTOR phosphorylation, suggesting that the mTOR signaling pathway may be involved in CENPO-mediated regulation of LUAD development.

**Conclusions:**

In pan-cancer, especially LUAD, CENPO may be a potential biomarker and oncogene. Furthermore, CENPO has been implicated in immune cell infiltration in pan-cancer and represents a potential immunotherapeutic target for tumor therapy.

**Supplementary Information:**

The online version contains supplementary material available at 10.1186/s12931-023-02408-3.

## Background

Cancer is one of the leading causes of death and severe disability that affects the quality of life in countries around the world, and to date there are currently no absolute cures for cancer [1]. A Coronavirus disease 2019 (COVID-19) hampered cancer diagnosis and treatment in 2020. For example, limited access to care because of health care setting closures leads to delays in diagnosis and treatment that can result in a short-term decline in cancer incidence followed by a rise in disease progression and ultimately higher mortality. Despite major advances in cancer therapy, including immunotherapy, targeted therapy, and radiation therapy, the 5-year overall survival (OS) remains unsatisfactory for patients [2]. Nowadays, immunotherapy has made huge success in cancer treatment, especially immune checkpoint blocking therapy [3, 4]. With the continuous development and refinement of public databases such as The Cancer Genome Atlas (TCGA), it is more convenient to further discover new immunotherapy targets by performing pan-cancer analysis of a single gene and assessing the correlations with clinical prognosis, immune infiltration and related signaling pathways. Therefore, it is essential to develop a novel cancer diagnostic and prognostic biomarker.

Cancer is a complex disease in which tumors interact with the immune system. The tumor microenvironment (TME) consists of several cells with a large proportion of infiltrating immune cells [5]. TME plays a crucial role in the origin and development of human cancers. Previous studies have shown that normal expression of centromeric proteins is critical for mitosis [6] and that abnormal nucleocentromeric proteins increase the incidence of chromosomal instability and aneuploidy, leading to tumorigenesis [7]. CENPO, an important member of the centromere protein (CENP) family, facilitates the premature separation of sister chromatids during recovery from cell death-related spindle damage [8]. Deletion of the CENPO protein results in aneuploidy and aneuploidy chromosomal gains, which can lead to the development of disease or cancer [9]. Recent studies have shown that the alteration of CENPO expression has been found to contribute to the development of gastric cancer and colorectal cancer [10, 11]. The mechanism of CENPO in bladder cancer may involve mitosis and complement as well as coagulation cascade signaling pathways [12]. CENPO can prevent the separation of sister chromatids and cell death after spindle injury, and may play a promoting role in colorectal cancer through the epithelial mesenchymal transition (EMT) and PI3K/AKT signaling pathway [11]. A member of its family, CENPA, may be a prognostic biomarker for lung adenocarcinomas (LUADs) [13]. Furthermore, CENPL has be proven to been associated with immune cell infiltration in pan-cancer, offering a potential target for immunotherapy [14]. Today, it is even more important to look for immunorelated biomarkers today because of the huge success of immunotherapy. However, there is still a lack of correlation between CENPO and immune infiltration. Therefore, we conducted an in-depth analysis and evaluated the potential value of CENPO in the prognosis of cancer and immune response, particularly in LUAD.

This research aimed to investigate the correlation between CENPO expression and immunity and visualize its prognostic landscape in pan-cancer, especially in LUAD. There is still a high mortality rate for lung adenocarcinoma (LUAD) worldwide [2]. In addition to enrichment analysis of CENPO coexpression genes, we also investigated the association between CENPO and immune infiltration. Furthermore, in vitro and in vivo experiments based on LUAD cells validated our bioinformatics results. Together, our results suggest that CENPO may serve as an oncogene and immunoinfiltration-related biomarker in pancancer, especially LUAD.

## Materials and methods

### Data processing

RNA sequencing, somatic mutation, phenotype data, and related clinical data were downloaded from Sangerbox database (http://vip.sangerbox.com/login.html), which contains 34 types of human cancer, and TCGA (https://portal.gdc.cancer.gov/), which contains 33 types of cancer. Detailed analysis procedures can be found in the Additional file 1: Material S1.

### Expression analysis of CENPO

Using the TCGA combined with the Genotype Tissue Expression (GTEx) cohort, CENPO expression levels were assessed in 34 normal tissues, 21 tumor cell lines, and 34 tumors. Log2 transformation was performed on the expression data, and two groups of *t-*tests were performed on these tumor types; *P* < 0.05 was the difference in expression between tumor tissue and normal tissue. Data analysis uses R software, and the box plots were drawn using the R package ‘ggpubr’. The relationship between CENPO and cancer stage was analyzed using the “Stage plots” module of GEPIA (gepia2.cancer-pku.cn/#index). Furthermore, tumor mutation burden (TMB), microsatellite instability (MSI), and mutant-allele tumor heterogeneity (MATH) data were downloaded that was only available from the TCGA database. TMB was determined by counting the number of insertion or deletion events in repetitive gene sequences, MSI was determined by counting the overall mutation occurrences per million base pair, and MATH was determined by all mutant-allele frequencies.

### Diagnostic and prognostic analysis

Using the data from the TCGA database, the potential value of CENPO in cancer diagnosis was assessed with ROC curves. AUC > 0.5 was considered to be of high diagnostic value. The potential value of CENPO in cancer prognosis was evaluated using overall survival (OS) and disease-free survival (DFS) from the GEPIA database.

### Mutant personality analysis

The mutational signatures of CENPO in different tumors were analyzed using the cBioPortal tool (http://www.cbioportal.org/). Entering “CENPO” in the “Query” module through the “TCGA Pan Cancer Atlas Studies” cohort, the location, type and amount of CENPO modifications can be found in the “cancer type summary” and “mutation” module. The association between CENPO mutations and clinical outcomes was derived from the module of “comparison / survival”. Databases of somatic mutations and somatic copy number alternations (CNAs) were obtained from TCGA datasets. The CNAs correlated with the expression of CENPO, and the threshold copy number at the alteration peaks was analyzed by GISTIC 2.0 (https://cloud.genepattern.org/). The patients were divided into the first 25% CENPO^high^ (n = 214**)** and the last 25% CENPO^low^ (n = 225) groups according to the expression value of CENPO. The maftools package was also used in the R software to download and visualize the somatic mutations of patients with CENPO^high^ and CENPO^low^ in the TCGA-LUAD databases.

### Functional and enrichment analysis

LinkedOmics (www.linkedomics.org/login.php) was used to detect the coexpression of CENPO in the “HiSeq RNA” platform and the ‘TCGA_LUAD’ cohorts. Pearson’s test was used to assess the correlation between CENPO and its coexpressed genes. Using single-cell sequence data from the “correlation plot” module of the CancerSEA website (biocc.hrbmu.edu.cn/CancerSEA/home.jsp), the correlation between CENPO and functional states of 18 cancer types was analyzed. The biological functions and pathways of CENPO were analyzed by the gene set variation analysis (GSVA) for gene ontology (GO) terms and the gene set enrichment analysis (GSEA) for Kyoto Encyclopedia of Genes and Genomes database (KEGG) terms and HALLMARK terms.

### Immunofiltration analysis

The extent of immune cell infiltration in 32 tumor types was assessed using the “GENE” module based on the TIMER database. Through the TISIDB tool (cis.hku.hk/TISIDB/index.php), the associations of CENPO with chemokines, chemokine receptors, and major histocompatibility complexes (MHC) were analyzed. The relationships between CENPO expression and ESTIMATE score and tumor infiltration immune cells (TIICs) in multiple tumors were explored via the SangerBox website, including B cells, CD4 + T memory cells, CD8 + T cells, NK cells, monocytes, macrophages, neutrophils, etc. The relationships between CENPO expression and microsatellite instability (MSI), tumor mutation burden (TMB), and mutant-allele tumor heterogeneity (MATH) in different tumors from TCGA cohorts were investigated via the SangerBox website. Pearson’s rank correlation test was performed and the partial correlation (cor) and the P-value were generated.

### CENPO-ceRNA regulatory network analysis

The CENPO targeting miRNAs were predicted by several target gene prediction programs, consisting of miRWalk (http://mirwalk.umm.uni-heidelberg.de/), miRDB (http://mirdb.org/), miRabel, and TargetScan (http://www.targetscan.org/vert_72/). Only the 82 predicted miRNAs that appeared in these four programs were included for subsequent analyses. Then, differently expressed miRNAs (DE-miRNAs) were selected according to the TCGA database and only 43 DE-miRNAs were significantly associated with the survival of LUAD patients. Only miR-370 was at the interstation of these miRNA sets. In addition, upstream target lncRNAs of miR-370 were predicted and analyzed via LncBase database (http://carolina.imis.athena-innovation.gr/diana_tools/web/index.php?r=lncba-sev2%2Findex). Subsequently, Cytoscape software was applied to visualize the lncRNA-miRNA-CENPO regulatory network.

### Patients and tissue samples

Total of 75 cases of LUAD tissues were obtained from patients hospitalized in the First Affiliated Hospital of Kunming Medical University, and no patients received radiotherapy or chemotherapy before surgical excision. The histopathological characteristics of all LUAD patients were independently diagnosed by two pathologists (Table 1). This study was approved by the Ethics Committee of the First Affiliated Hospital of Kunming Medical University and conformed to the ethical principles established by the Declaration of Helsinki. Written informed consent was signed and obtained from all participants.

**Table 1:** Relationship between CENPO expression and tumor characteristics in patients with lung cancer

Features	No. of patients	CENPO expression		p value
		Low	High	
All patients	75	39	36	
Age (years)				0.100
≤ 62	39	17	22	
> 62	35	22	13	
Gender				0.343
Male	69	37	32	
Female	6	2	4	
Tumor size				0.132
< 5 cm	36	22	14	
≥ 5 cm	39	17	22	
Lymph node positive				0.073
= 0	46	28	18	
> 0	28	11	17	
Grade				0.247
I	2	1	1	
II	53	30	23	
III	20	8	12	
Stage				0.001**
1	28	21	7	
2	25	12	13	
3	22	6	16	
T Infiltrate				0.220
T1	7	3	4	
T2	51	25	26	
T3	16	10	6	
T4	1	1	0	
Lymphatic metastasis (N)				0.821
N0	46	28	18	
N1	11	7	4	
N2	6	3	3	

**P < 0.01

### Immunohistochemistry (IHC) staining

LUAD tissues and normal tissues were frozen and cut into 4–8 μm sections at room temperature for 30 min. IHC staining is performed as previously described [15]. Briefly, sections were incubated overnight at 4° C with primary antibody against CENPO (1:200, Biorbyt, USA, #orb335144). Subsequently, sections were incubated with a specific secondary antibody for 2 h at room temperature, with horseradish peroxidase (HRP) conjugated streptavidin for 1 h, stained with diaminobenzidine (DAB) for 5 min and stained with hematoxylin. Immunohistochemical staining results were blindly evaluated by two experienced pathologists. Five fields of visual were randomly selected from each section, and the CENPO positive expression in LUAD tissues was detected under a light microscope with a color of brownish yellow.

### Cell lines and cell culture

Two human adenocarcinoma cell lines (A549 and NCI-H1299) were purchased from the BeNa biological Co., Ltd. (BNCC, Beijing, China) and cultured in Dulbecco’s modified Eagle medium (DMEM) supplemented with 10% fetal bovine serum (FBS) (Gibco). Cells were cultured in a humidified incubator at 37° C with 5% CO_2_.

### Lentiviral shCENPO construction

Small hairpin RNAs (shRNAs) were designed to target human CENPO genes by Shanghai GeneChem Co. Ltd. (Shanghai, China). Lentivirus without shRNA insert was used as a control. The shCENPO fragments were inserted into the lentivirus vector and transfected into 293T cells with packaging vectors using Lipofectamine 3000 (Invitrogen) as the soon as cell density reached 70%. After the construction of the lentivirus, cells were transfected with lentivirus to establish CENPO knockdown A549 or NCI-H1299 cells. After 48 h of transfection, the recombinant lentivirus was collected from cell culture medium for further infection. The knockdown effect was confirmed with western blot.

### RNA isolation and RT-qPCR

Total RNA was extracted with TRIzol reagent (Invitrogen). cDNA was synthesized using the PrimeScript RT Reagent Kit (TaKaRa). RT-qPCR was performed with SYBR Premix Ex Taq (TaKaRa) on the 7900HT Fast Real-Time PCR System (Applied Biosystems). The primers used are shown as follows: CENPO, forward, 5′-CCAGCCGAGGAGTTTGTGTC-3′ and reverse, 5′-CCGGAGTGGTTTCTGTATGAC-3′; GAPDH, forward, 5′-TGACTTCAACAGCGACACCCA-3 ′ and reverse, 5′-CACCCTGTTGCTGTAGCCAAA-3′. Relative mRNA expression (CENPO/GAPDH) was determined using the comparative Ct method (2^−ΔΔCt^).

### Western blotting analysis

The total protein of cells transfected with lentiviruses was lysed in RIPA lysis buffer (Beyotime, Shanghai, China) supplemented with protease inhibitor cocktail (Roche Applied Science) and the protein concentration was measured using the BCA Protein Assay Kit (Beyotime). Protein lysates (20 µg) were separated by 12% SDS-PAGE and transferred to PVDF membranes. The membranes were then blocked with 5% non-fat milk for 1 h at room temperature before being incubated with primary antibodies overnight at 4° C. The protein was then co-incubated with the goat anti-rabbit IgG polyclonal antibody (1:3000) labeled with horseradish peroxidase (HRP) at room temperature for 2 h. Finally, the protein signal was visualized using the chemiluminescence ECL-PLUS kit (Thermo Fisher Scientific).

### Celigo cell counting assay

After digesting A549 or NCI-H1299 cells with trypsin, cells were resuspended into cell suspension and counted with a counting plate (Cellometer, Cat. #SD-100). Cells were cultured into 6-well plates at a density of 2 × 10^3^ cells/well and the culture system was 100 μL/ well. The Celigo Imaging Cytometer (Nexcelom) was monitored once a day for five consecutive days. The number of cells was accurately calculated according to the number of EGFP-positive cells in each scanning orifice. The 5-day data were calculated and the cell proliferation curve was plotted.

### Transwell assays

Transwell assays were performed in transwell chambers (8-μm pores, BD Biosciences) inserted in 24-well plates with Matrigel. The upper chambers were coated with 100 μl serum-free medium containing 1 × 10^5^ cells, while the lower compartment contained 500 μl culture medium supplemented with 20% FBS. After incubating at 37 °C for 24 h, cells that had migrated to the lower surface of the membrane were fixed with 4% paraformaldehyde for 30 min, stained with 0.1% crystal violet for 20 min. Five visual fields were randomly selected and observed under a light microscope (200×), and images were captured for enumeration.

### Wound-healing assay

Cells transfected with Lentivirus A549 or NCI-H1299 were inoculated in 6-well plates (100 μL/well) at a density of 5 × 10^4^ cells/well for 5 days. In summary, vertical lines for each well were drawn at 0 h, 24 h, and 48 h using a pipette. After incubation, cells were washed with PBS, fixed with 3.7% paraformaldehyde (Corning) for 15 min, and stained with 1% crystal violet (Corning) for 10 min. Finally, cells were placed under a microscope for image acquisition and Image J software (National Institutes of Health) was used to quantify the distance (μm) between the scratches at different time points.

### Flow cytometry assay for cell cycle and apoptosis

Apoptosis was quantified with annexin V-FITC and propidium iodide (PI). Lentivirus-transfected (shCtrl and shCENPO) cells were harvested, rinsed twice with PBS and resuspended in 1 × binding buffer. Before detection by flow cytometry, resuspended cells were stained with FITC Annexin V for 25 min and PI for 15 min in the dark at room temperature. The results of apoptosis rate were analyzed using FlowJo X 10.0.7 R2 software. For cell cycle analysis, treated cells were centrifuged for 5 min and then were successively washed and precipitated by PBS and 1 × binding buffer, fixed with 70% ethanol for at least 1 h, stained with PI (Sigma). The monitored proportion of cells in the G1, S and G2 phases of the shCtrl and shCENPO groups were detected by flow cytomotry.

### Tumor formation assay in nude mice

All animal experiments were carried out with the approval of the Animal Care and Use Committee of The First Affiliated Hospital of Kunming Medical University. Female BALB/c nude mice (4–5 weeks old) kept in a pathogen-free facility. A549 cells transfected with shCtrl and shCENPO were harvested, washed, and resuspended in PBS. Next, 5 × 10^6^ cells in 150 μl PBS were subcutaneously injected into the left armpit of each mouse. With 10 mice per group, tumorigenesis rates in the shCtrl group and the shCENPO group were 50% and 20%, respectively. Mice were anesthetized with 0.7% sodium pentobarbital (10 μL/g) and placed in the IVIS spectral fluorescence imaging system (emission wavelength of 510 nm) to assess tumor burden. 39 days after injection, the mice were sacrificed and dissected, the tumors were photographed, weighed, and tumor volumes were calculated using the equation: length × width^2^ × 0.5. Finally, the expression of Ki67 (1: 200, Abcam, USA, #ab16667) was detected by IHC staining in mouse tumor tissues of shCENPO group and shCtrl group as previous described. Images were observed under a microscope (Nikon, Tokyo, Japan).

### Statistical analysis

All bioinformatics statistical analyses were performed by using R version 4.1.0. Each experiment was repeated at least 3 times and are expressed as mean ± standard deviation (SD). Statistical analysis was performed using GraphPad Prism 8.0 software (GraphPad Software Inc., San Diego, CA, USA). Significant differences between two groups were analyzed using Student's t-test. Analysis of variance analysis was used for the comparison of multiple groups, and a Dunnett test was used as a post hoc test. Pearson's chi-squared test was used to compare the clinicopathologic features. *P* < 0.05 were considered statistically significant.

## Results

### Up-regulation of CENPO in various human cancers

**Fig. 1:**
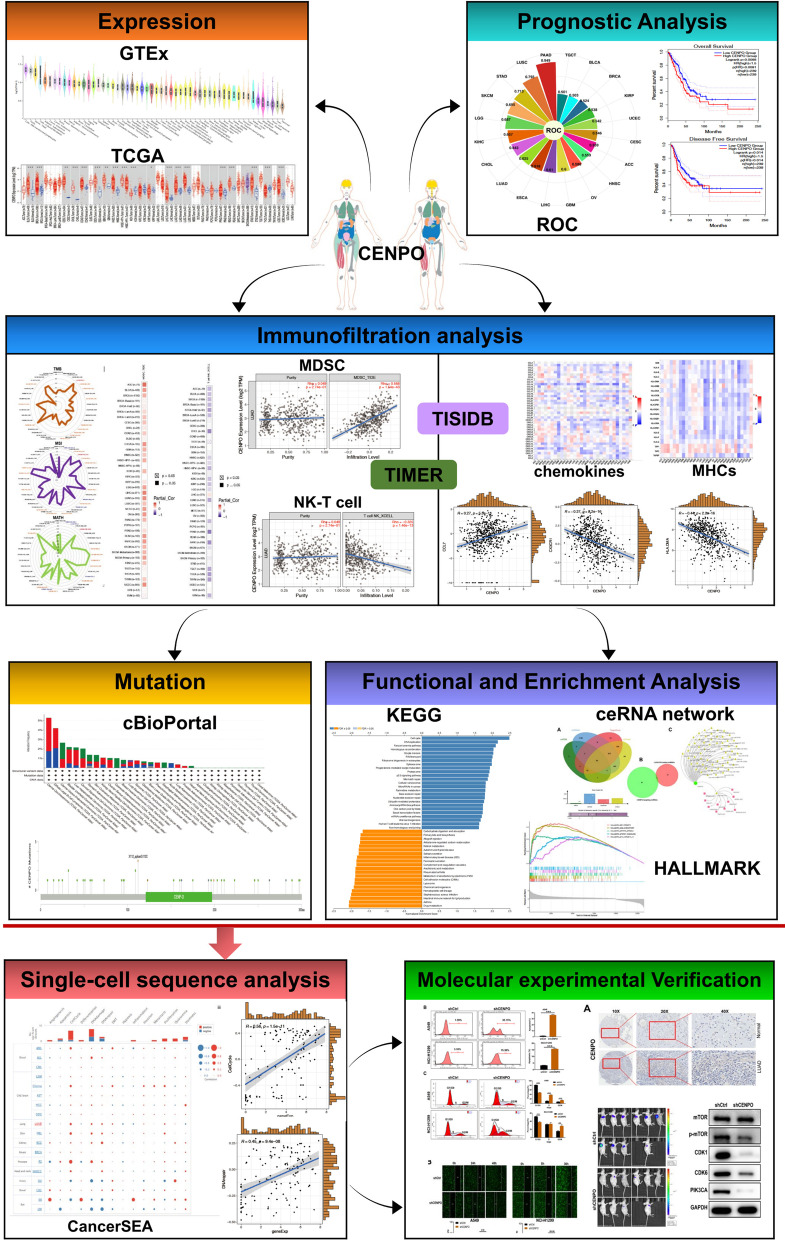
The flow chart of the entire study

**Fig. 2:**
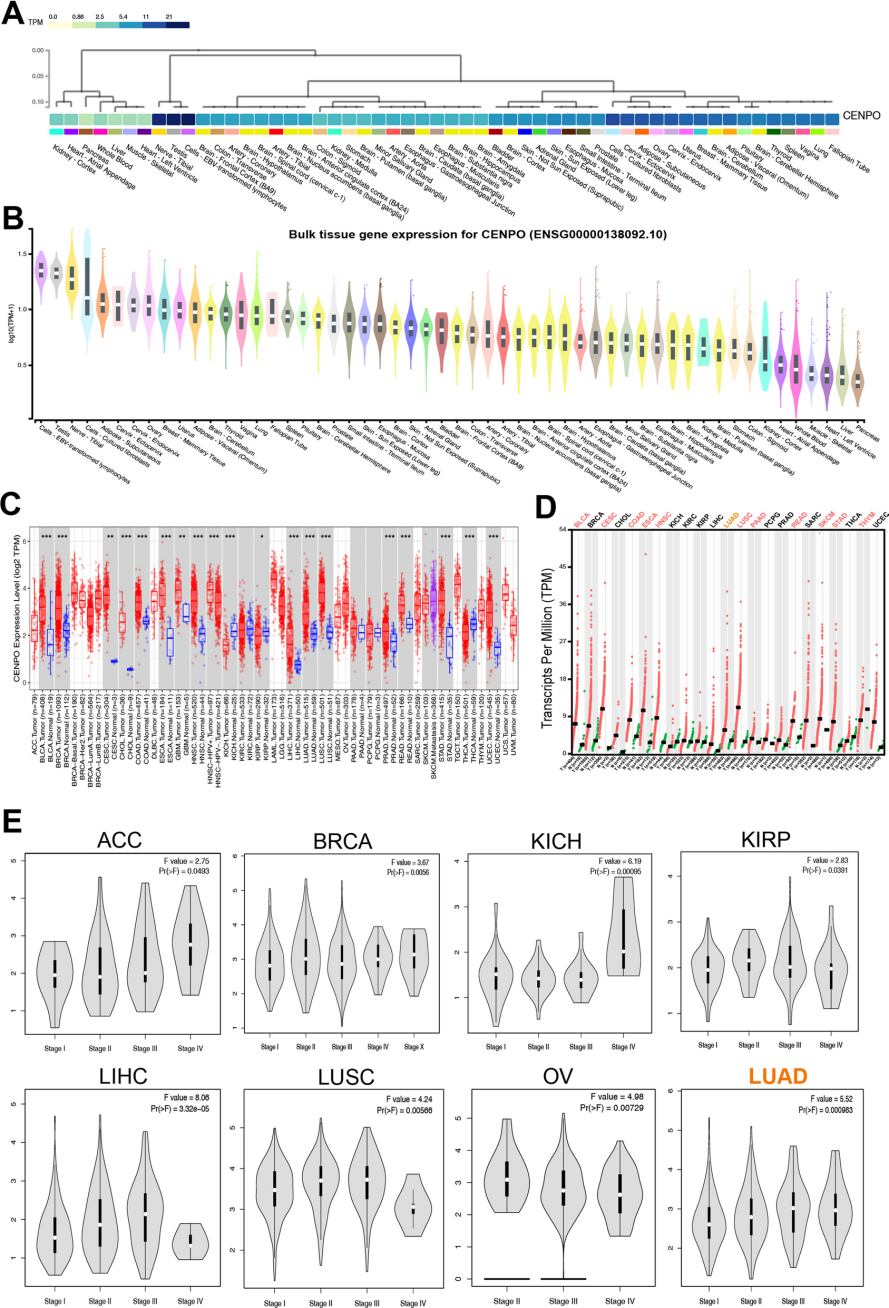
Expression profiles of CENPO in normal tissues and pancancers. **A** Expression levels of CENPO in various tissues and cells, and **B** bulk tissue gene expression for CENPO based on the GTEx database. **C** The expression of CENPO in the TIMER 2.0 database. **D** Comparison of CENPO expression between tumor and paired normal samples based on the TCGA database. **E** The correlation between CENPO expression and tumor stage (*p < 0.05, **p < 0.01, ***p < 0.001)

The flowchart of this study is presented in Fig. 1. Firstly, the expression levels of CENPO were analyzed in various tissues and cells in the GTEx database. In most other normal human tissues, the expression level of CENPO mRNA was detectable (Fig. 2A). The expression of large tissue genes for CENPO were higher in the normal human EBV transformed lymphocytes, Testis, Nerve-Tibial, Cultured fibroblasts, subcutaneous adipose tissue, cerevix, Ovary (log10 (TPM + 1) > 1); Fig. 2B). Moreover, Additional file 2: Fig. S1 showed the example of the CENPO differential splicing of gene in different tissues as well as its tissues specificity. The Sangerbox database was used to study the expression level of CENPO in pancancer. It could be observed that CENPO was up-regulated in most human cancers, including glioblastoma multiforme (GBM), lower grade glioma (LGG), uterine corpus endometrial carcinoma (UCEC), lung adenocarcinoma (LUAD), bladder urothelial carcinoma (BLCA), breast invasive cancer (BRCA), cervical and endocervical cancer (CESC), cholangiocarcinoma (CHOL), colon adenocarcinoma (COAD), esophageal carcinoma (ESCA), head and neck squamous cell carcinoma (HNSC), liver hepatocellular carcinoma (LIHC), serous cystadenocarcinoma (OV), lung squamous cell carcinoma (LUSC), stomach adenocarcinoma (STAD) and thyroid carcinoma (LAML), etc. (Additional file 2: Fig. S2A). In contrast, CENPO was downregulated in thyroid carcinoma (THCA) and kidney chromophobe (KICH) (Additional file 2: Fig. S2A). To further explore the expression level of CENPO, we analyzed CENPO expression in the TCGA datasets using the TIMER 2.0 database. The results obtained show that CENPO was up-regulated in most cancers (Fig. 2C), including BLCA, BRCA, CESC, CHOL, COAD, ESCA, GBM, HNSC, LIHC, LUAD, LUSC, prostate adenocarcinoma (PRAD), READ, STAD, and UCEC. However, CENPO was also negatively regulated in KICH, THCA, and kidney renal papillary cell carcinoma (KIRP) (Fig. 2C). In addition, a notable increase in CENPO expression in 23 types of cancer, respectively, in paired tumor samples compared to the corresponding normal samples (Fig. 2D). Next, we analyze the association between CENPO and the stages of pancancers on the GEPIA2 website. CENPO was significantly associated with the stage of adrenocortical carcinoma (ACC), BRCA, KICH, KIRP, LIHC, LUSC, OV and LUAD (Fig. 2E). These results suggest that CENPO expression is upregulated in various types of cancer, indicating that CENPO may play a potentially crucial role in cancer diagnosis.

### The diagnostic and prognostic role of CENPO in pan-cancer

**Fig. 3:**
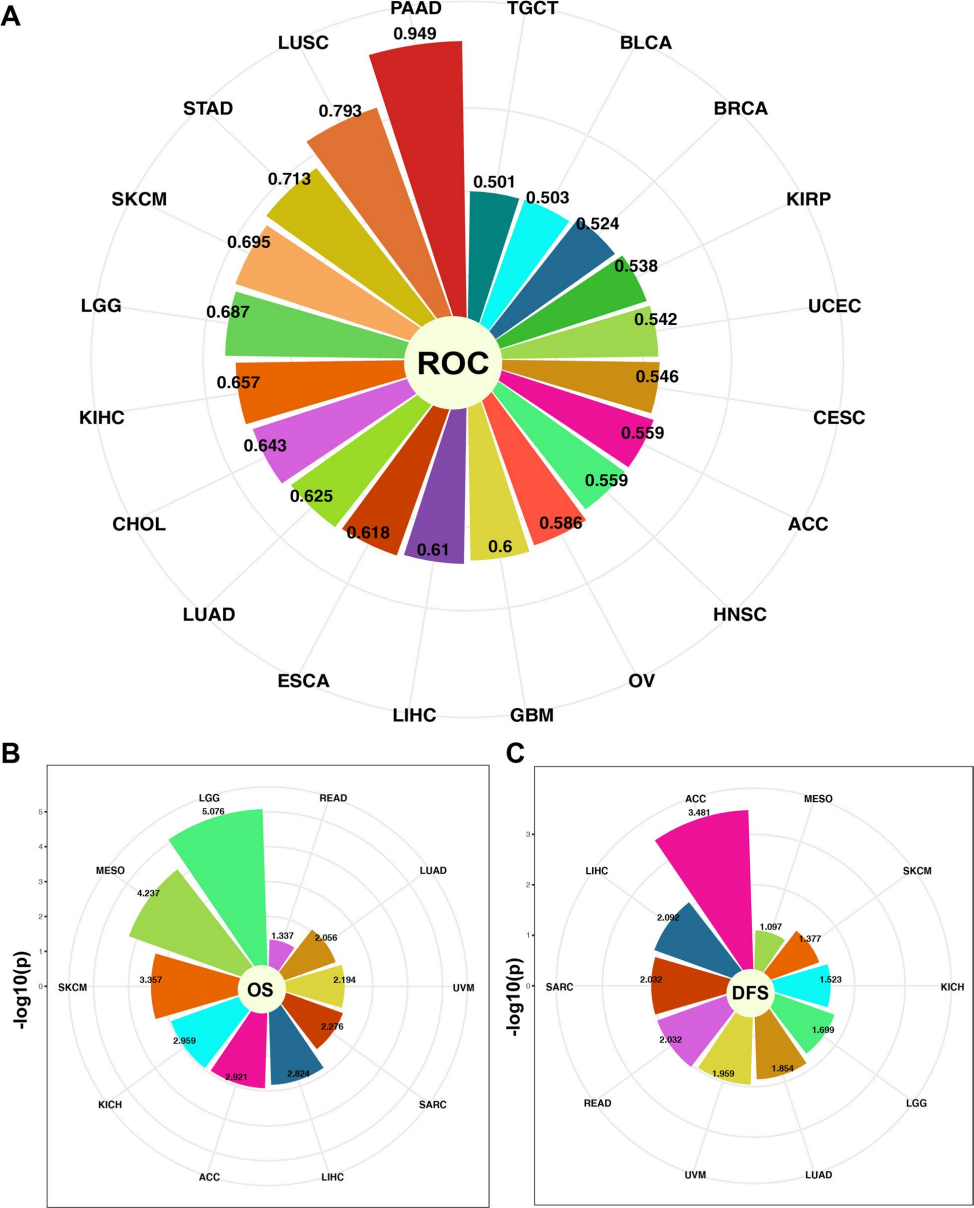
The diagnostic and prognostic value of CENPO in pancancer. **A** Circular histogram of the receiver operator characteristic (ROC) curves of CENPO in 16 cancers. Circular histogram of overall survival (OS) (**B**), and disease-free survival (DFS) (**C**,) of CENPO in 10 types of cancer using the Kaplan–Meier analysis

Subsequently, we evaluated the diagnostic value of CENPO in multiple types of cancers using ROC curves (Additional file 3: Table S1). As shown in Fig. 3A and Additional file 2: Fig. S2B, CENPO may act as a perfect diagnostic marker in ACC (AUC = 0.559), BLCA (AUC = 0.503), BRCA (AUC = 0.524), CESC (AUC = 0.546), CHOL (AUC = 0.643), ESCA (AUC = 0.618), GBM (AUC = 0.600), HNSC (AUC = 0.559), KICH (AUC = 0.657), UCEC (AUC = 0.542), KIRP (AUC = 0.538), LGG (AUC = 0.687), LIHC (AUC = 0.610), LUAD (AUC = 0.625), LUSC (AUC = 0.793), and OV (AUC = 0.586). Furthermore, an increase in CENPO level was associated with poor overall survival (OS) in ACC (p = 0.0012), KICH (p = 0.0011), LIHC (p = 0.0015), LUAD (p = 0.0088), sarcoma (SARC; p = 0.0053), uveal melanoma (UVM; p = 0.0064), LGG (p = 8.4e-06), SKCM (p = 0.00041) and mesothelioma (MESO; p = 5.8e-05) (Fig. 3B and Additional file 2: Fig. S3A). By contrast, increased CENPO expression correlates with better OS in READ (p = 0.046) (Fig. 3B and Additional file 2: Fig. S3A). Moreover, DFS results also showed that increased CENPO expression was associated with poorer prognosis in ACC (p = 0.00033), KICH (p = 0.03), LIHC (p = 0.0081), LUAD (p = 0.014), SARC (p = 0.0093), UVM (p = 0.011), LGG (p = 0.02), SKCM (p = 0.042), and MESO (p = 0.08) (Fig. 3C and Additional file 2: Fig. S3B).

### CENPO participates in immune infiltration of pan-cancer

**Fig. 4:**
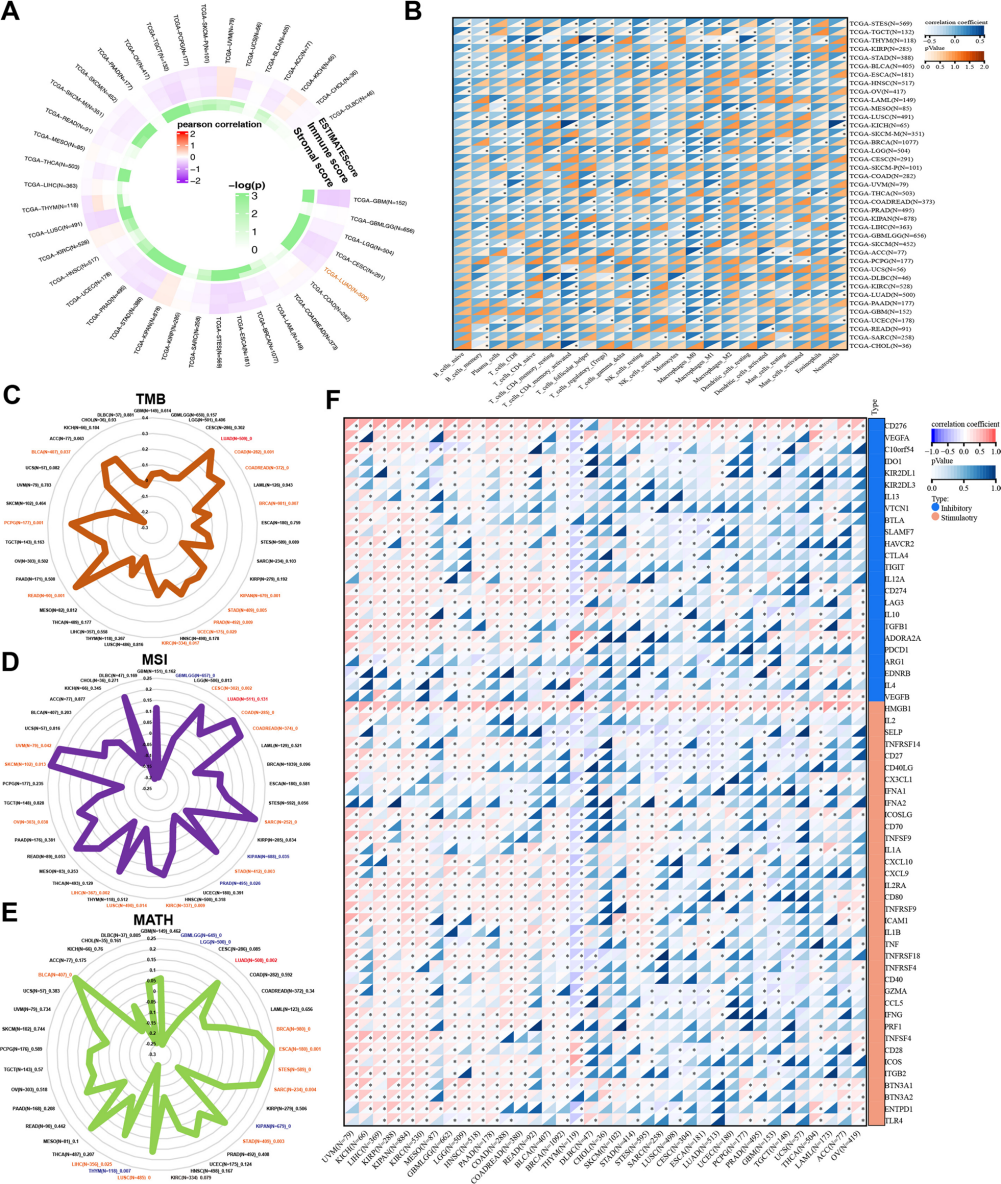
Relationship between CENPO expression and MSI, TMB, MATH, and immune checkpoints in pancancer. **A** The correlations between the ESTIMATE scores (ESTIMATE Score, Immune Score, and Stromal Score) and CENPO expression were analyzed in various tumors using the Sangerbox portal. **B** The relationship between CENPO expression and immune cell infiltration level was analyzed in various tumors by Sangerbox online website. Relationship between CENPO mRNA expression and **C** TMB, **D** MSI, and **E** MATH displayed by the radar chart. **F** Relationship between CENPO expression and immune checkpoints in multiple cancers. *P < 0.05, **P < 0.01, ***P < 0.001, ****P < 0.0001

First, we evaluated the correlation between ESTIMATE scores (ESTIMATE, immune, and stromal scores) and CENPO expression levels in pancancers. The immune score reflects the proportion of infiltrated immune cells in tumor tissues; stromal score reflects the proportion of stromal cells in the tumor tissues. ESTIMATE score is the sum of immune and stromal scores and reflects the status of the tumor immune microenvironment and tumor purity. Our results demonstrated a negative correlation between CENPO expression and ESTIMATE, immune, and stromal scores in ACC, GBM, CESC, LUAD, ESCA, STAD, TGCT, and LUSC (Fig. 4A and Additional file 4: Table S2). This suggested that high CENPO expression was associated with decreased infiltration of immune and stromal cells in several tumors, thereby resulting in high tumor purity. Conversely, CENPO expression showed positive correlation with ESTIMATE, immune, and stromal scores in KIPAN, KIRC, UVM, and KICH (Fig. 4A and Additional file 4: Table S2). Then we investigated the relationship between CENPO expression and TIIC levels in various tumors using the Sangerbox database. The relationship between CENPO expression and immune cell varies greatly in each type of tumor depending on the type of immune cell. The types of immune cells included memory B cells, plasma cells, CD8 + T cells, memory CD4 + and CD8 + T cells, NK cells, regulatory T cells, monocytes, macrophages, dendritic cells, mast cells, eosinophils, neutrophils (Fig. 4B and Additional file 5: Table S3). Furthermore, we investigated the relationship between CENPO expression levels and TMB,

MSI or MATH to determine if CENPO was a predictor of immunotherapeutic responses in multiple cancer types. The expression of CENPO showed positive relationship with TMB in LUAD (P = 0), COAD (P = 0.001), READ (P = 0), BRCA (P = 0.007), KIPAN (P = 0.001), STAD (P = 0.005), PRAD (P = 0.009), UCEC (P = 0.029), KIRC (P = 0.017), READ (P = 0.001) PCPG (P = 0.001), and BLCA (P = 0.037) (Fig. 4C and Additional file 6: Table S4). Furthermore, the expression of CENPO showed positive correlation with MSI in CESC (P = 0.002), COAD (P = 0, READ (P = 0), SARC (P = 0), STAD (P = 0.003), KIRC (P = 0.009), LUSC (P = 0.014), LIHC (P = 0.002), OV (P = 0.038), SKCM (P = 0.013) and UVM (P = 0.042), while negative association with MSI in GBML (P = 0), KIPAN (P = 0.035) and PRAD (P = 0.026) (Fig. 4D and Additional file 6: Table S4). The expression of CENPO also showed positive correlation with MATH in LUAD (P = 0.002), BRCA (P = 0), ESCA (P = 0.001), SARC (0.004), STAD (P = 0.003), LUSC (P = 0), LIHC (P = 0.025) and BLCA (P = 0), while a negative association with MATH in GBML (P = 0), LGG (P = 0), KIPAN (P = 0), THYM (P = 0.007) (Fig. 4E and Additional file 6: Table S4). Immune checkpoint (ICP) blockade proteins are the most promising targets of cancer immunotherapeutic treatments. Therefore, we analyzed the relationship between expression levels of ICP genes and CENPO in multiple cancer types. The expression of CENPO showed a positive correlation with ICP genes in most cancers, such as UVM, KICH, KIRP, KIPANKIRC, GBM, HNSC, and BRCA. However, CENPO expression showed a negative correlation with most ICP genes in THYM (Fig. 4F and Additional file 7: Table S5 and Additional file 8: Table S6). In summary, CENPO plays a vital role in immune infiltrates in pancancer and might act as a novel immunotherapy target in tumor therapy.

**Fig. 5:**
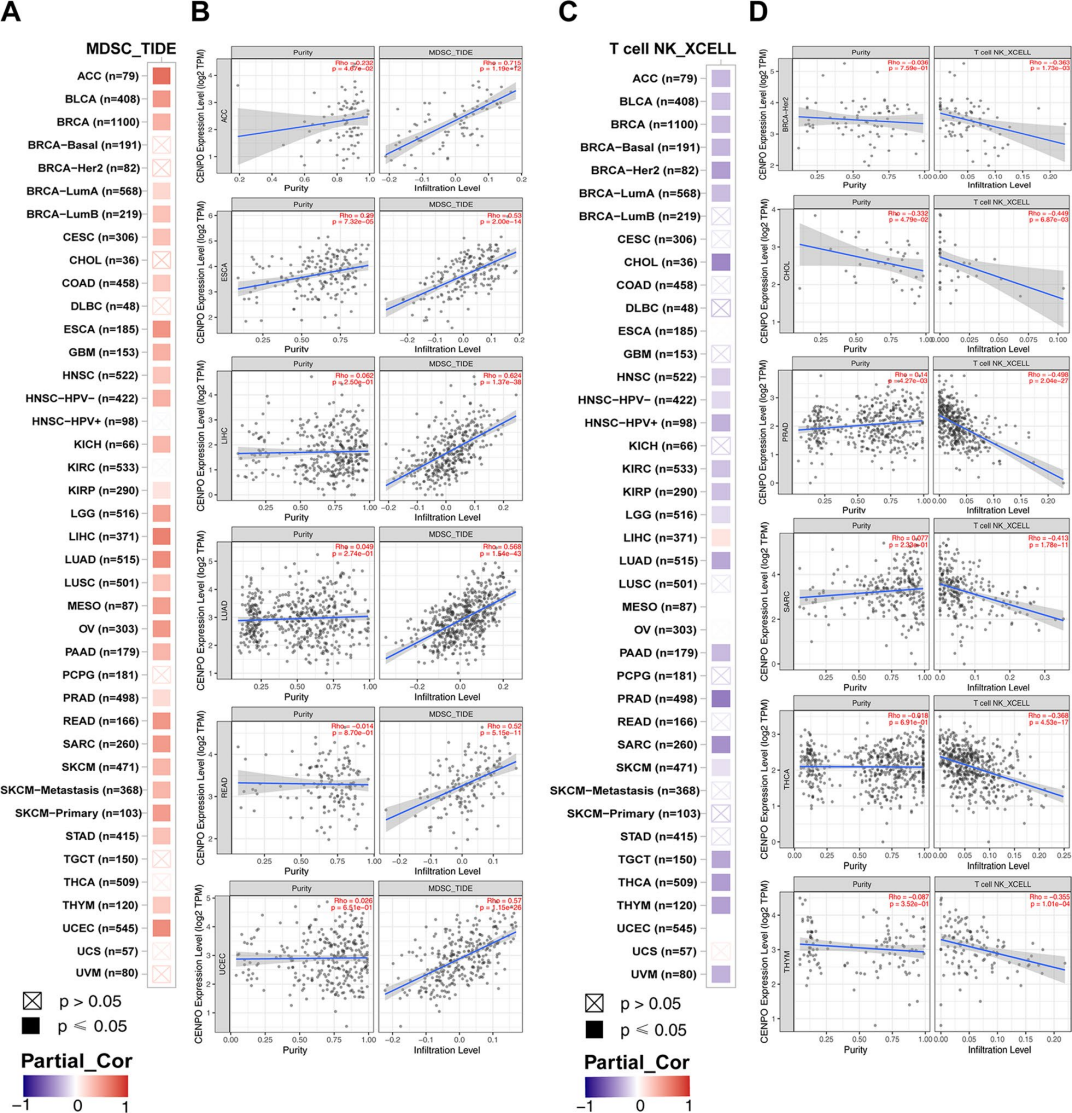
The association between CENPO expression and immune cell infiltration. **A**, **B** CENPO expression is positively associated with MDSC infiltration in pan-cancer. **C**, **D** CENPO expression is negatively associated with NK-T cell infiltration in pan-cancer

Subsequently, the potential role of CENPO in immune cell infiltration was investigated using TIMER 2.0. The results showed that the level of myeloid-derived suppressor cell (MDSC) infiltration was significantly positively correlated with CENPO expression in most of the tumors (Fig. 5A); the top 6 tumors were ACC (Rho = 0.715, p = 1.19e−12), ESCA (Rho = 0.53, p = 2.00e−14), LIHC (Rho = 0.624, p = 1.37e−38), LUAD (Rho = 0.568, p = 1.54e−43), READ (Rho = 0.52, p = 5.15e−11), and UCEC (Rho = 0.57, p = 1.15e−26) (Fig. 5B). Interestingly, the results showed that a negative association between the infiltration level of T-cell NK and CENPO expression in most of the tumors (Fig. 5C); the top 6 tumors were BRCA-Her2 (Rho = − 0.363, p = 1.73e−03), CHOL (Rho = − 0.449, p = 6.87e−03), PRAD (Rho =  − 0.498, p = 2.04e−27), SARC (Rho =  − 0.413, p = 1.78e−11), THCA (Rho =  − 0.368, p = 4.53e−17), and THYM (Rho =  − 0.355, p = 1.01e−04) (Fig.5D). In addition, we studied the association between CENPO and cell infiltration of several immune cells in LUAD. There was a negative association between CENPO and the level of T-cell NK (Rho = 0.375, p = 1.46e−13), CD4 + T cells (Rho = 0.136, p = 2.52e−03), CD8 + T cells (Rho = 0.176, p = 9.74e−05), Treg (Rho = 0.104, p = 2.06e−02), B cells (Rho = 0.224, p = 7.35e−08), Myeloid dendritic cell (Rho = 0.333, p = 1.35e−14), Monocyte (Rho = 0.116, p = 9.97e−03) and Macrophage M2 (Rho = 0.256, p = 4.27e−10) (Additional file 2: Fig. S4).

**Fig. 6:**
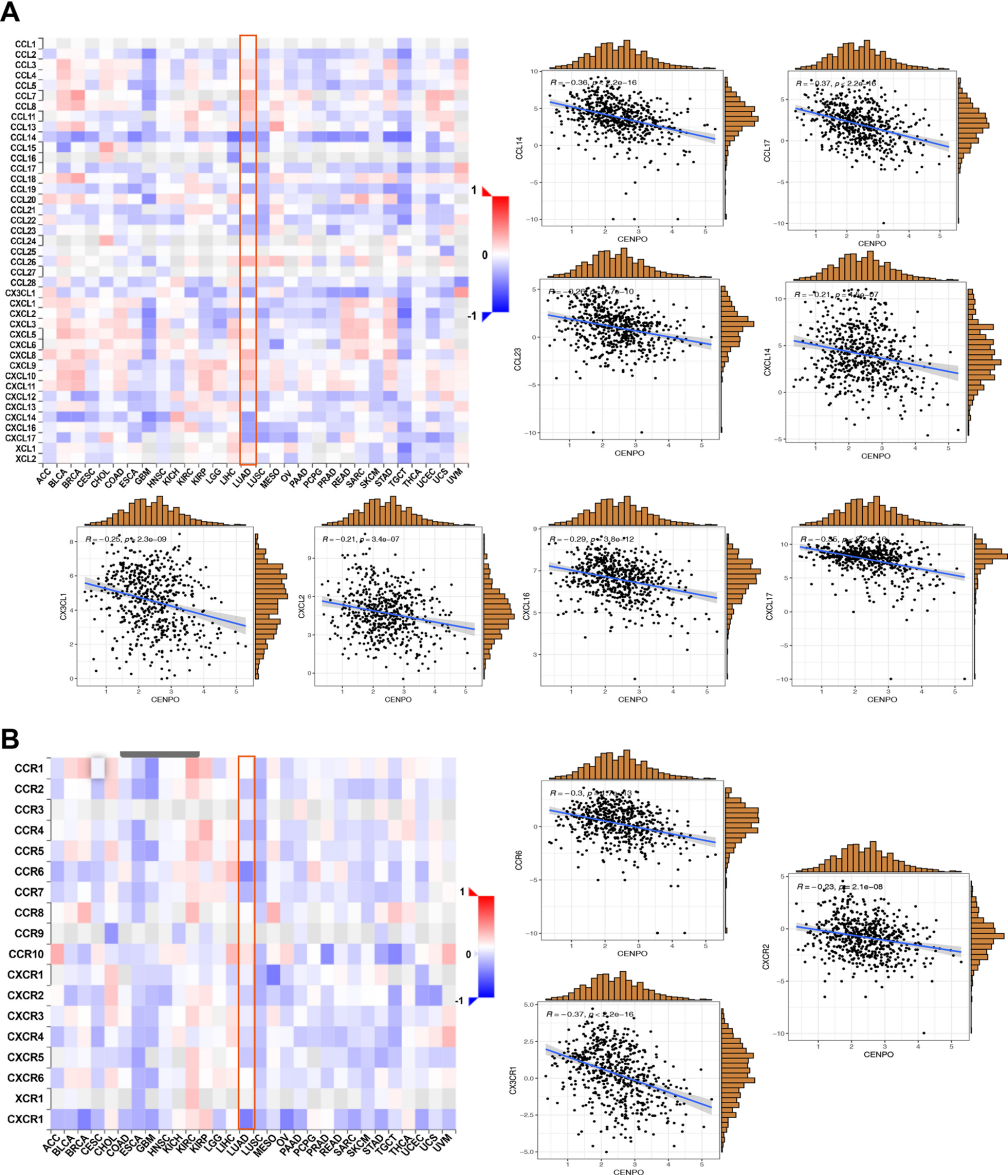
Correlation between CENPO expression and chemokines and chemokine receptors in the TISIDB database. **A** The expression of CENPO is negatively associated with most chemokines in pan-cancer. **B** The expression of CENPO is negatively associated with most chemokine receptors in pan-cancer

We then further analyzed the association of CENPO with chemokines and chemokine receptors through the TISIDB database and found a negative correlation between the expression of CENPO and most of chemokines (Fig. 6A). Regarding LUAD, the top eight chemokines were CCL14 (R = 0.36, p < 2.2e−16), CCL17 (R = 0.37, p < 2.2e−16), CCL23 (R = 0.26, p = 1.7e−10), CXCL14 (R = 0.21, p = 4.5e−07), CX3CL1 (R = 0.25, p = 2.3e−09), CXCL2 (R =  − 0.21, p = 3.4e−07), CXCL16 (R =  − 0.29, p = 3.8e−12), and CXCL17 (R = 0.35, p < 2.2e−16). Figure 6B showed the correlations of 18 types of chemokine receptors with CENPO expression and observed that CENPO was negatively associated with three chemokine receptors in LUAD, including CCR6 (R = 0.3, p = 1.7e−13), CX3CR1 (R = 0.37, p < 2.2e−16), and CXCR2 (R = 0.23, p = 2.1e−08). Furthermore, our results also suggested that CENPO expression is negatively associated with most of the major histocompatibility complexes (MHC) (Additional file 2: Fig. S5A). Regarding LUAD, CENPO expression is negatively correlated with 12 types of MHCs, including B2M (R = 0.2, p = 1.4e−06), HLA-DMA (R = 0.44, p < 2.2e−16), HLA-DMB (R = 0.31, p = 2.6 e−14), HLA-DOA (R = 0.27, p = 6.1e−11), HLA-DOB (R = 0.26, p = 1.2e−10), HLA-DPA1 (R = 0.33, p = 2.4e−16), HLA-DPB1 (R = 0.29, p < 2.2e−16), HLA-DQA1 (R = 0.22, p = 1.3e−07), HLA-DQB1 (R =  − 0.26, p < 1.6e−10), HLA-DRA (R =  − 0.39, p < 2.2e−16), HLA-DRB1 (R = 0.38, p < 2.2 e−16), and HLA-E (R =  − 0.29, p = 6.3e−13) (Additional file 2: Fig. S5B).

### Characteristics of CENPO mutations in LUAD

**Fig. 7:**
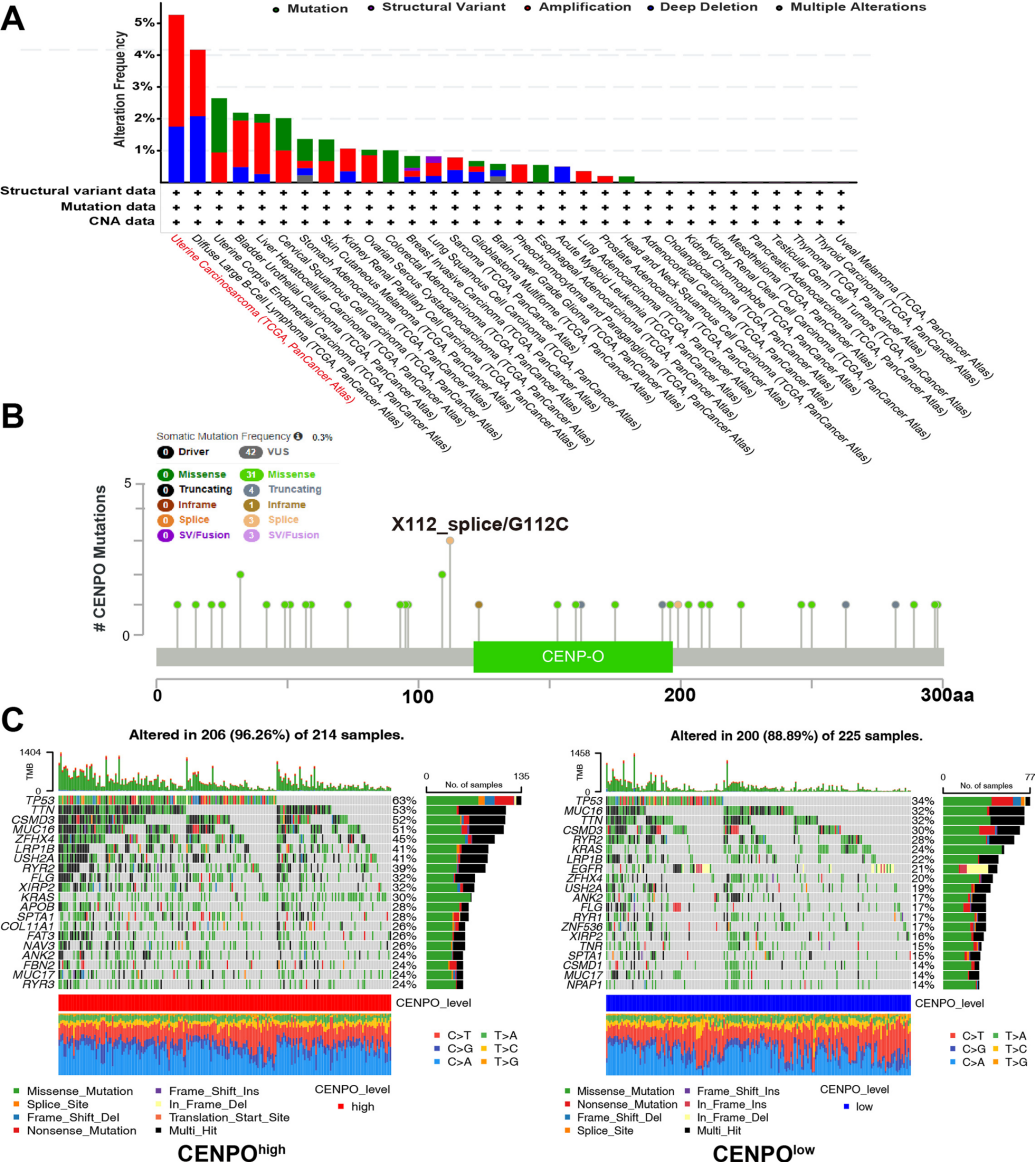
The mutation character of CENPO in pancancer. **A** Alteration frequency of CENPO in pan-cancer. **B** The subtypes and distributions of CENPO somatic mutations. **C** The waterfall plots of differential somatic mutations in LUAD, including the CENPO^high^ group and the CENPO^low^ group

Next, we analyzed the CENPO mutation status in the TCGA cohorts. As shown in Fig. 7A, CENPO alteration was observed in 32 cancers, of which uterine carcinoma had the highest incidence rate of 5.34%. Figure 7B also showed the sites, types, and numbers of CENPO mutations. The relationship between CENPO expression and specific genomic characteristics such as somatic mutations and copy number variations (CNVs) was then analyzed in the TCGA-LUAD dataset. The CENPO^high^ group (n = 214) showed a high frequency of somatic mutations in the genes TP53 (63%), TTN (53%), CSMD3 (52%), MUC16 (51%) and ZFHX4 (45%) and the CENPO^low^ group (n = 225) showed high frequency of mutations in the TP53 (34%), MUC16 (32%), TTN (32%), CSMD3 (30%) and RYR2 (28%) (Figs. 7C and Additional file 2: Fig. S6A). Furthermore, we analyzed the association between the CENPO mutation and the clinical outcomes of different cancers. Additional file 2: Fig. S6B indicated that breast invasive carcinoma patients with CENPO mutation had poor prognosis in OS (p = 1.718e-3), but not DFS (p = 0.184), DSS (p = 0.722), and PFS (p = 372).

### The function analysis of CENPO in LUAD

**Fig. 8:**
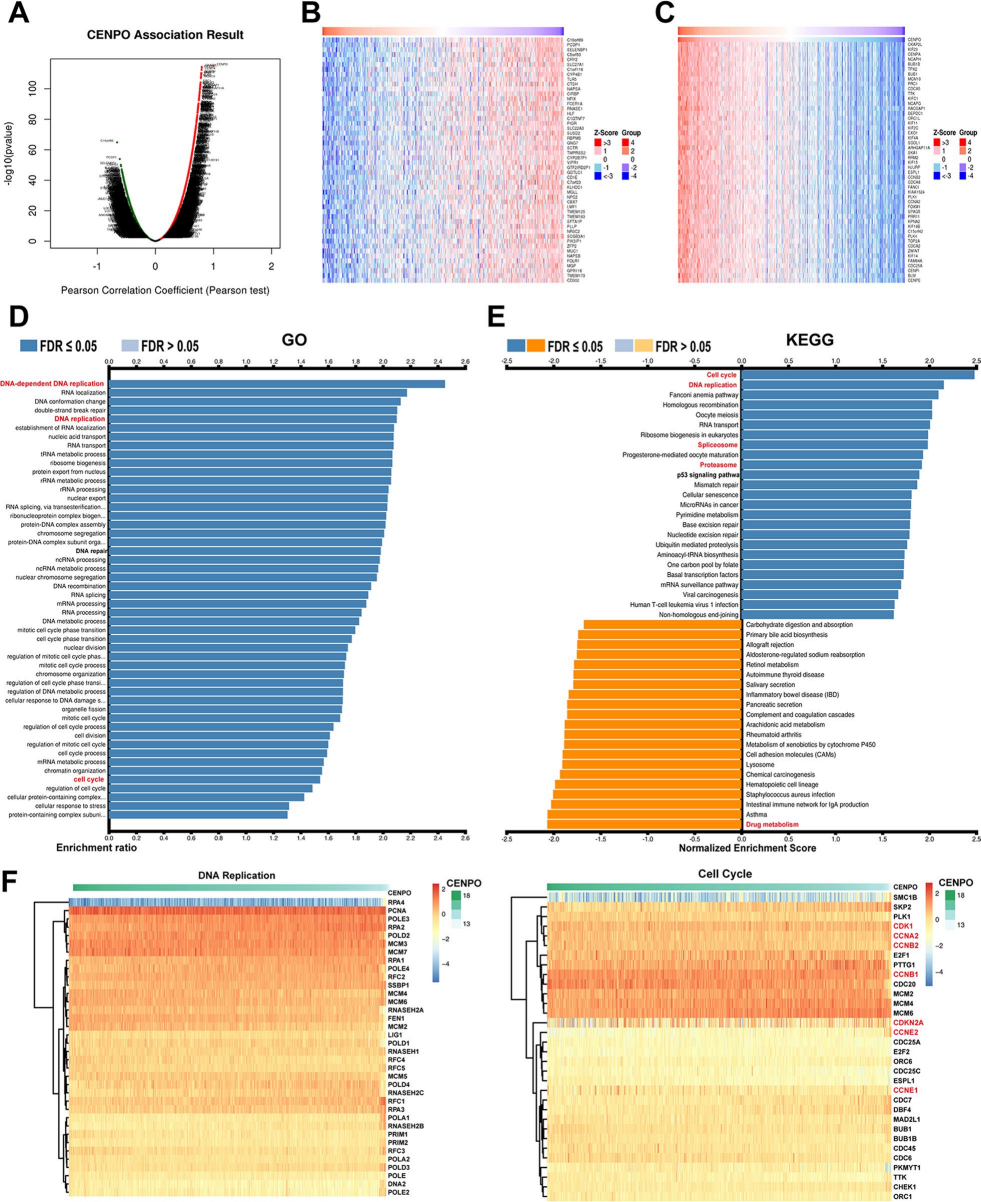
The enrichment analysis of CENPO co-expression genes in LUAD. **A** The CENPO co-expression genes in LUAD. **B**, **C** The top 50 genes were positively and negatively correlated with CENPO. **D**, **E** GO and KEGG analysis of co-expression genes of CENPO in the LUAD cohort. **F** Heat maps of the correlation between CENPO and the DNA replication and cell cycle

We further explore the biological function of CENPO in LUAD through the LinkedOmics database. Figure 8A suggests the positively and negatively related genes with CENPO. The top 50 genes are shown in Fig. 8B and C. Moreover, GO analysis (Biological function) indicated that CENPO mainly involved in DNA-dependent DNA replication, DNA replication, cell cycle phase transition, cell cycle process, etc. (Fig. 8D). KEGG analysis presented enrichment in cell cycle, DNA replication, spliceosome, proteasome, drug metabolism, etc. (Fig. 8E). We further demonstrated the correlation of CENPO with the top two significantly enriched signaling pathways, including cell cycle and DNA replication, using a heatmap, where CENPO was significantly associated with cell cycle-related proteins, including CDK1, CCNA2, CCNB1, CCNB2, CCNEA, CCNE2, and CDKN2A (Fig. 8F). These evidences suggest that CENPO is likely to play a key role in cell cycle regulation of LUAD.

### Construction of the ceRNA network that regulates CENPO expression and interacting genes of CENPO in LUAD

**Fig. 9:**
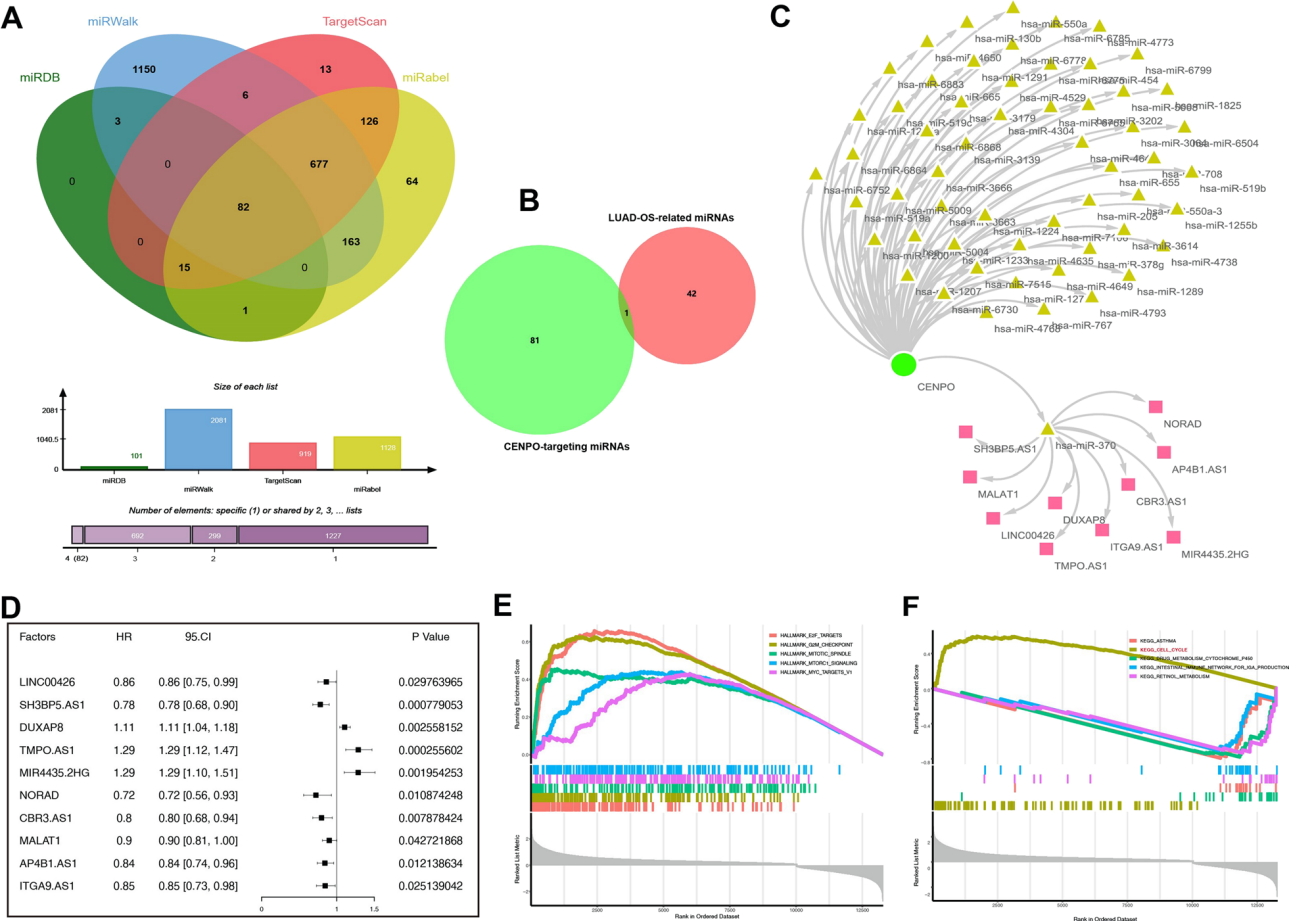
The regulatory network constructed in LUAD. **A** The upstream miRNAs of CENPO were predicted by miRDB, miRWalk, Targetscan and miRabel databases and the intersection was taken (82 intersection miRNAs). **B** Venn of 82 intersection miRNAs and 43 differently expressed miRNAs that were significantly related to the prognosis of patients with LUAD. **C** The Cytoscape-built regulatory network was constructed by Cytoscape, including CENPO, 82 miRNAs and 10 lncRNAs. **D** Survival analysis of 10 lncRNAs in the pancancer described by the forest plot. Functional enrichment pathways of CENPO based on the GSVA algorithm. **E** Top five positive enriched pathways based on the HALLMARK terms. **F** Four negative and one positive enriched pathway based on the KEGG terms

In recent years, several studies have shown that long non-coding RNAs (lncRNAs) play a significant role in tumorigenesis by regulating the expression of the downstream mRNAs through sequestering of their target miRNAs [16]. Therefore, we investigated the lncRNA-miRNA network that may regulate CENPO expression in LUAD. First, we selected the miRDB, miRWalk, Targetscan, and miRabel databases and identified 82 miRNAs that could target CENPO (Fig. 9A). We then analyze the miRNAs in LUAD that were significantly correlated with patient survival in TCGA-LUAD (P < 0.05) and found 43 miRNAs and only miR-370 at the intersection of the two sets (Fig. 9B). These results indicated that hsa-miR-370 could target CENPO expression in the LUAD. Next, we identified 61 validated lncRNAs that can target hsa-miR-370 using the LncBase database, of which only 10 lncRNAs were significantly associated with the prognosis of patients with LUAD (Fig. 9D). Total 83 miRNAs and 10 validated lncRNAs were used to construct a lncRNA-miRNA-CENPO regulatory network using the cytoscape software (Fig. 9C). These results demonstrated the upstream regulatory network that may regulate aberrant expression of CENPO in LUAD.

**Fig. 10:**
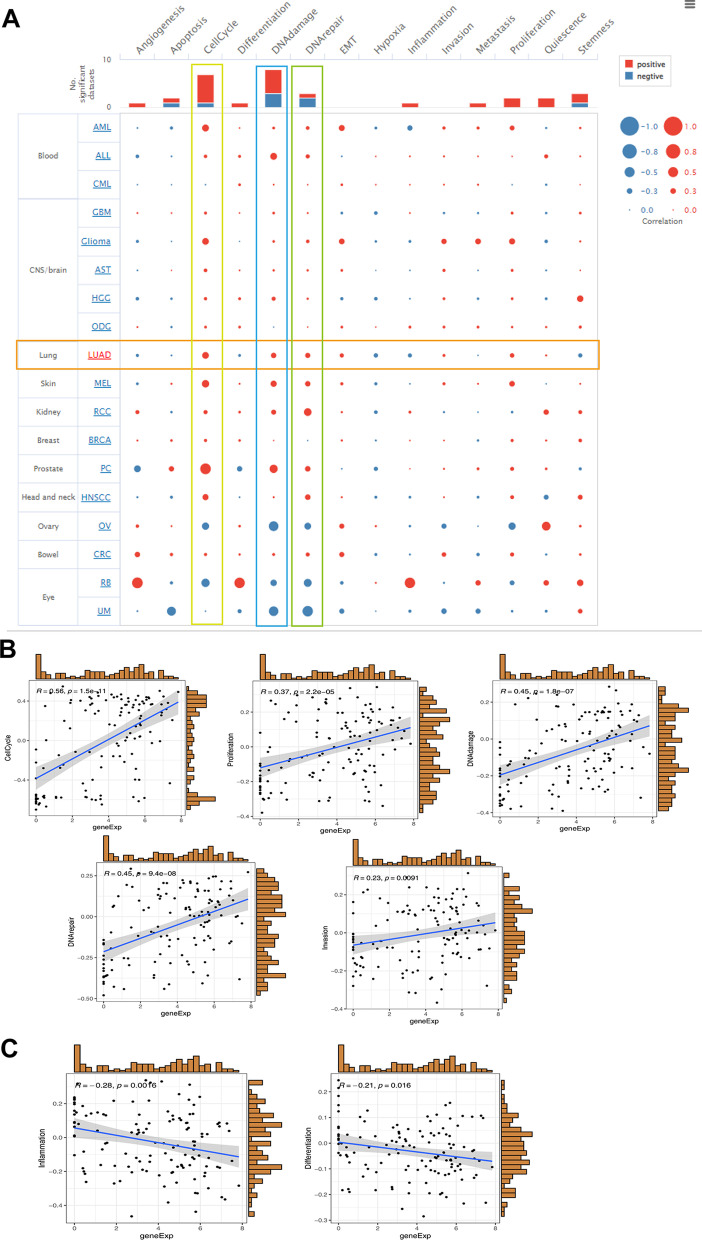
Correlation between CENPO expression and 18 cancer functional states using single-cell sequence data from the CancerSEA database. **A** The correlation between CENPO expression and 18 cancer functional states in pancancer. **B** CENPO expression is positively correlated with cell cycle, proliferation, DNA damage, DNA repair, and LUAD invasion. **C** The expression of CENPO is negatively correlated with the inflammation and differentiation of LUAD

Furthermore, we found that the top five positively enriched pathways were “E2F targets” ‘G2M checkpoint’, ‘mitotic spindle’, ‘mtorc1 signaling’ and “MYC targets” based on the HALLMARK (Fig. 9E). Meanwhile, we found that the top one positively enriched pathway was cell cycle, while the top four negatively enriched pathways were asthma, drug metabolism cytochrome P450, intestinal immune network for Ig A production and retinol metabolism based on the KEGG terms (Fig. 9F). We then analyzed the associated between CENPO and the functional states of 18 cancer using single cell sequence data in CancerSEA. CENPO was positively correlated with cell cycle, DNA damage, and DNA repair of most cancers (Fig. 10A). Interestingly, CENPO was inversely correlated with the hypoxia and angiogenesis of most cancers (Fig. 10A). In LUAD, CENPO expression was significantly positively correlated with cell cycle (R = 0.56, p = 1.5e−11), DNA damage (R = 0.45, p = 1.8E−07), DNA repair (R = 0.45, p = 9.4e−08), invasion (R = 0.23, p = 0.0091), proliferation (R = 0.37, p = 2.2e−05) (Fig. 10B), and negatively associated with inflammation (R = 0.28, p = 0.0016) and differentiation (R = 0.21, p = 0.016) (Fig. 10C).

### CENPO knockdown induced apoptosis and G2 arrest of LUAD

**Fig. 11:**
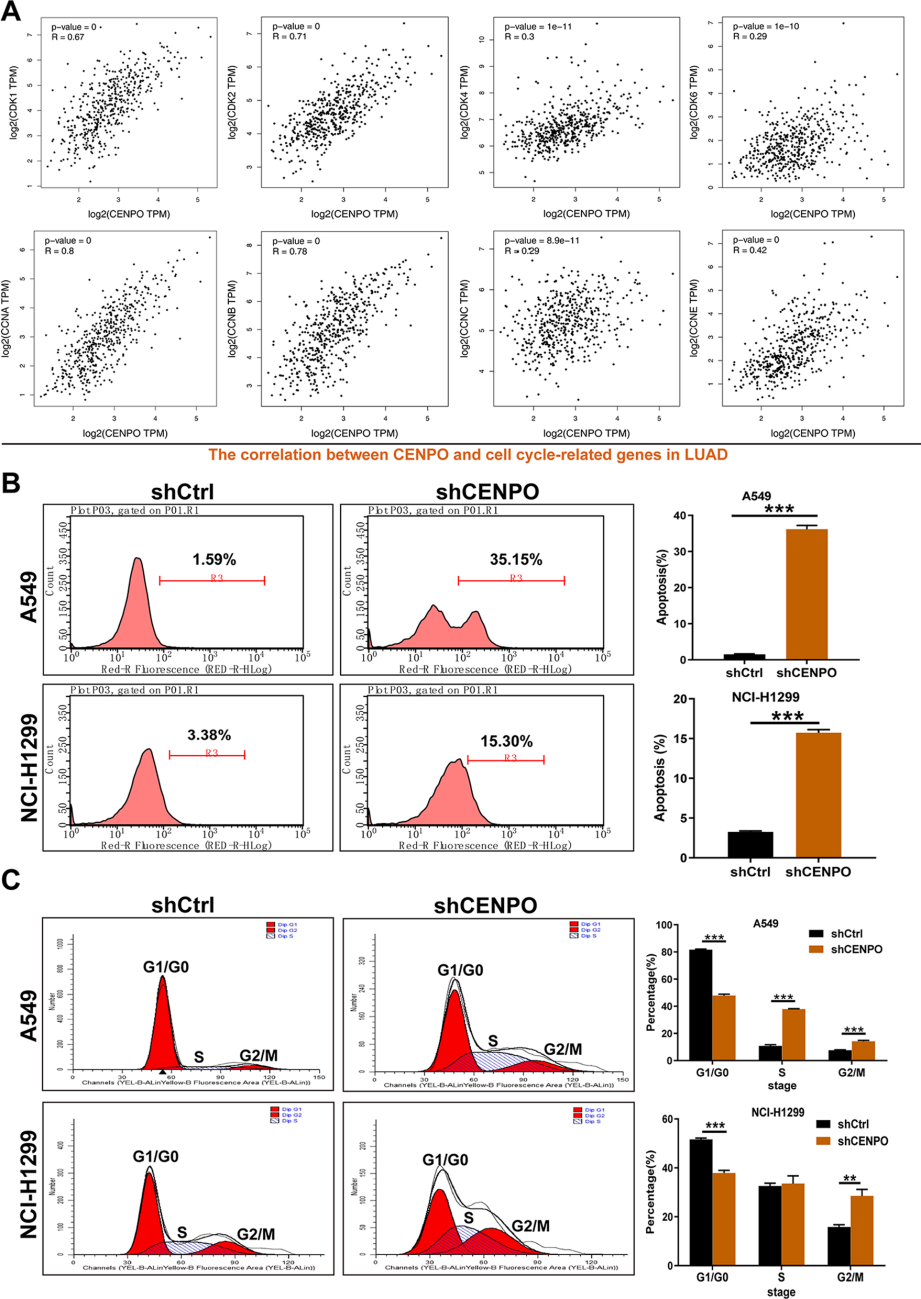
CENPO participates in the regulation of cell cycle of LUAD. **A** The correlation between CENPO expression and CDK1, CDK2, CDK4, CDK6, CCNA, CCNB, CCNC, and CCNE, in the GEPIA2.0 database. **B** Effects of down-regulation of CENPO expression on cell apoptosis of A549 and HCI-H1299 cells. **C** Flow cytometry with PI staining was used to detect the effect of CENPO knockdown on the cell cycle distribution of A549 and HCI-H1299 cells. The presented results were representative of experiments repeated at least three times. Data was presented as the mean ± SD (n = 3). *P < 0.05, **P < 0.01 and ***P < 0.001

We further confirm our bioinformatics results through in vitro and in vivo experiments. The analysis of the biological pathway in Fig. 8 and 9 shows that CENPO was significantly associated with the cell cycle in LUAD. Therefore, we further detected the association between the expression of the CENPO and the CDK family (CDK1/2/4/6), and the Cyclin family (CCNA/B/C/E) expression in GEPIA2.0. The results obtained show that CENPO was positively associated with CDK1 expression (R = 0.67, p = 0), CDK2 (R = 0.71, p = 0), CDK4 (R = 0.3, p = 1e−11), CDK6 (R = 0.29, p = 1e−10), CCNA (R = 0.8, p = 0), CCNB (R = 0.78, p = 0), CCNC (R = 0.29, p = 8.9e−11) and CCNE (R = 0.42, p = 0) (Fig. 11A). Furthermore, CENPO was also overexpressed in two LUAD cell lines (A549 and HCI-H1299) and we designed a shRNA to destroy CENPO in A549 and HCI-H1299 cells. The knockdown efficiency is presented in Additional file 2: Fig. S7A and B. The silence of CENPO significantly induced apoptosis (Fig. 11B) and arrest of G2 (Fig. 11C) of A549 and HCI-H1299 cells. Collectively, CENPO-knocked down LUAD cells could induce cell apoptosis and arrest cycle in G2/M phase.

### CENPO knockdown suppressed LUAD cell growth and metastasis via mTOR signaling pathway

**Fig. 12:**
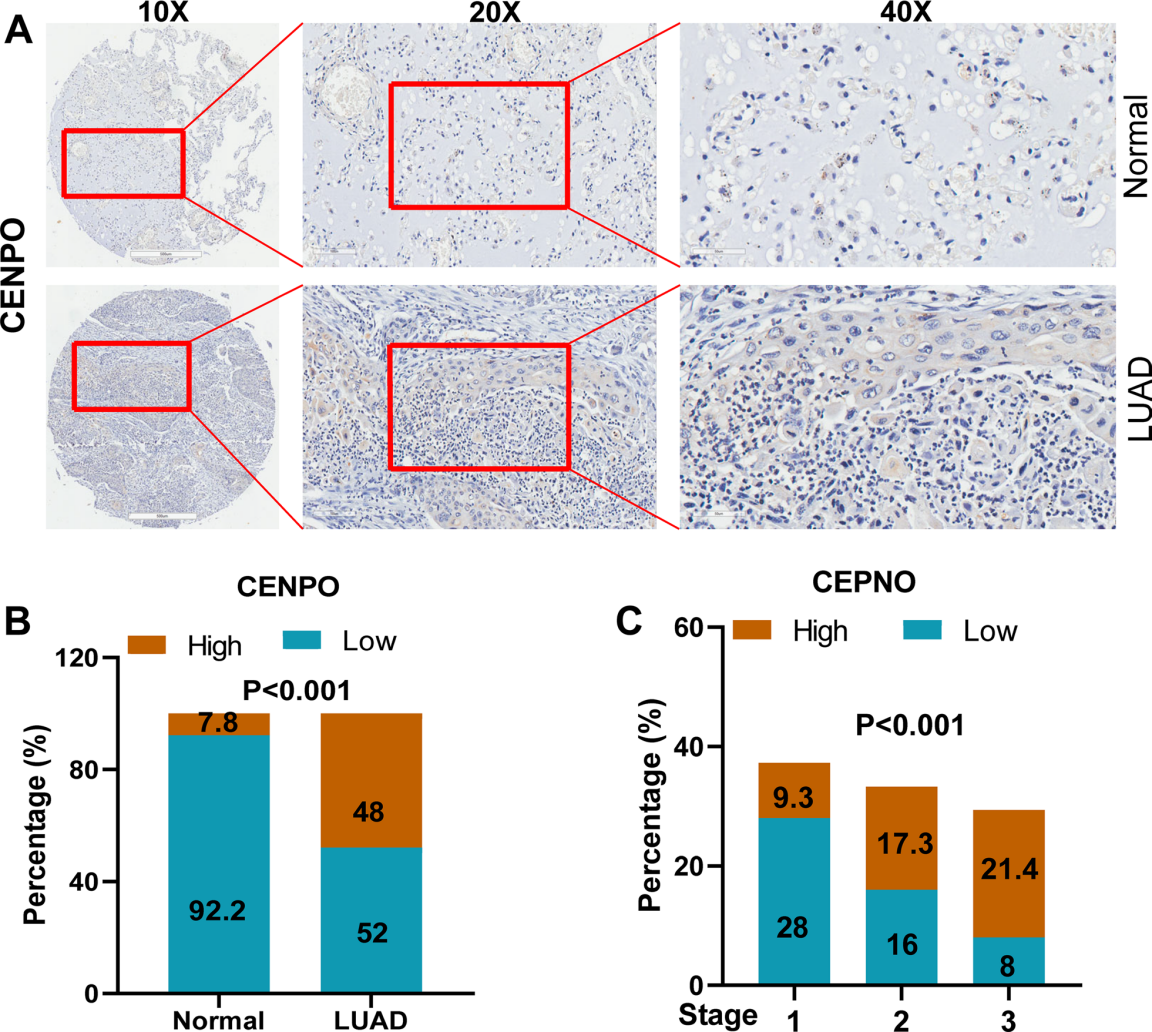
Validation of CENPO expression in LUAD. **A** The expression intensity of CENPO in LUAD tissues and the corresponding normal tissues was accomplished through IHC. **B** Expression patterns of CENPO in LUAD tissues and adjacent normal tissues revealed in immunohistochemistry analysis. **C** Expression patterns of CENPO in LUAD tissues with different tumor TNM stage

**Fig. 13:**
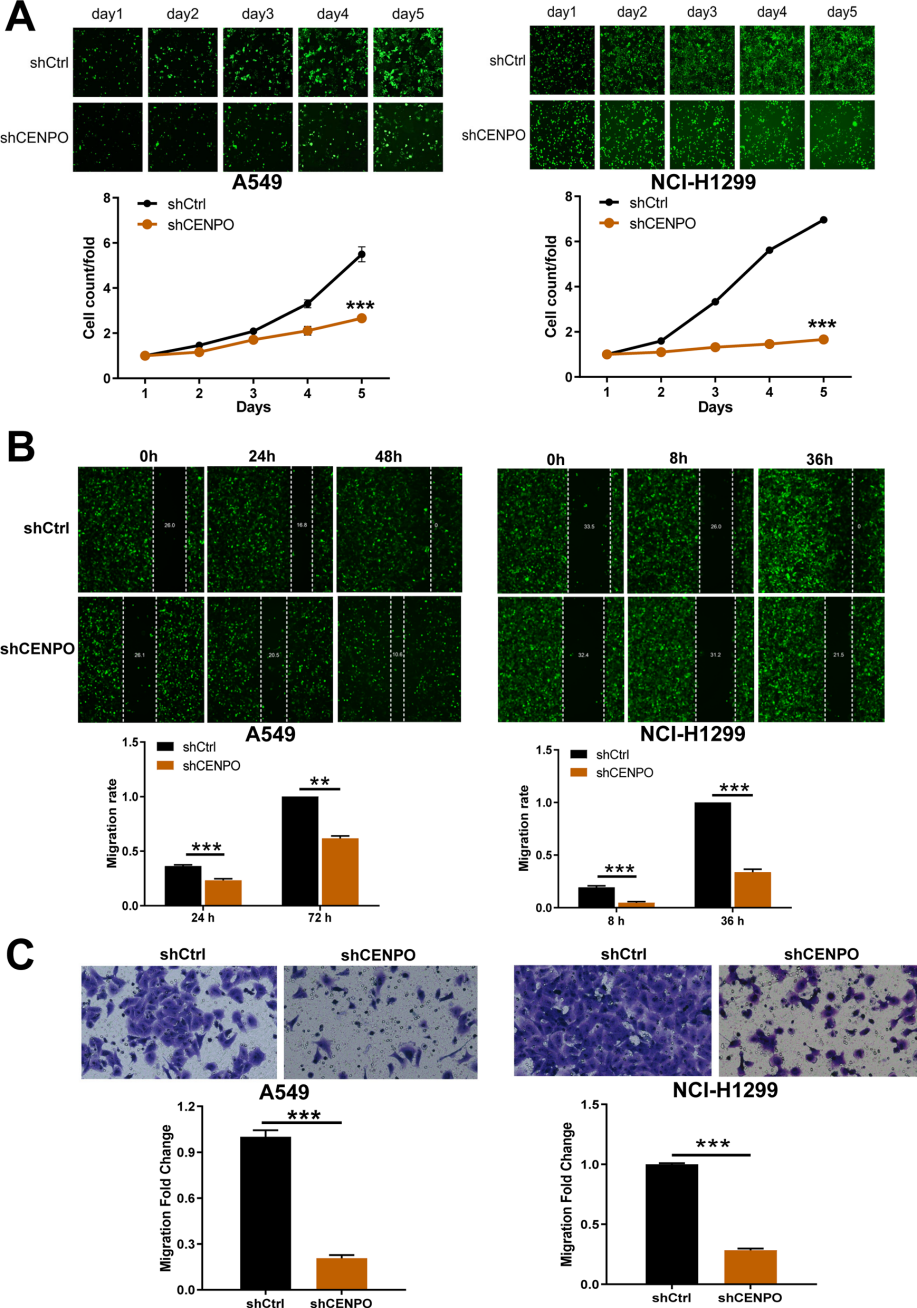
Knockdown of CENPO significantly suppressed proliferation, migration and invasion in LUAD cells. **A** Cell proliferation of A549 and HCI-H1299 cells transfected with shCENPO was assessed by Celigo staining assay. **B**, **C** The effect of CENPO knockdown on A549 and HCI-H1299 cell migration (**B**) and invasion (**C**) was evaluated using a wound healing assay (**B**) and a Transwell assay (**C**)

Next, we further investigated the function of CENPO on cell proliferation and metastasis. First, IHC staining showed that CENPO was significantly up-regulated in LUAD tissues (Fig. 12A). Moreover, Mann–Whitney U analysis indicated that there was a significant positive correlation between the CENPO expression and tumor stage (P = 0.001) (Table 1). High expression of CENPO was observed in 36 of 75 LUAD tissues (48%) and in 4 of 75 normal tissues (7.8%) (P < 0.001) (Fig. 12B and Table 2). Pearson correlation analysis further suggested that the high expression of CENPO may predict the deterioration of LUAD patients that the higher the tumor stage, the higher the proportion of patients with high levels of CENPO (Fig. 12C and Table 3). The results of the Celigo staining results (Fig. 13A) suggest that CENPO knockdown inhibited the proliferation of A549 and HCI-H1299 cells. Micrographs of the Transwell chamber showed significantly fewer cells in the shCENPO group than in the shCtrl group. As shown in Fig. 13B, the migration ability of A549 and HCI-H1299 cells in the shCENPO group within 48 h or 36 h decreased significantly, respectively, compared to the shCtrl group. The invasive cells per field specifically indicated that A549 and HCI-H1299 cells were significantly reduced (Fig. 13C).

**Table 2:** Expression patterns in LUAD tissues and para-carcinoma tissues revealed in immunohistochemistry analysis

CENPO expression	LUAD tissue		Para-carcinoma tissue		p value
	Cases	Percentage (%)	Cases	Percentage (%)	
Low	39	52.0	47	92.2	0.000***
High	36	48.0	4	7.8	

***P < 0.001

**Table 3:** Relationship between CENPO expression and tumor characteristics in patients with LUAD

	CENPO
Stage	
Pearson correlation	0.391
Significance (two-tailed)	0.001**
N	75

**P < 0.01

**Fig. 14:**
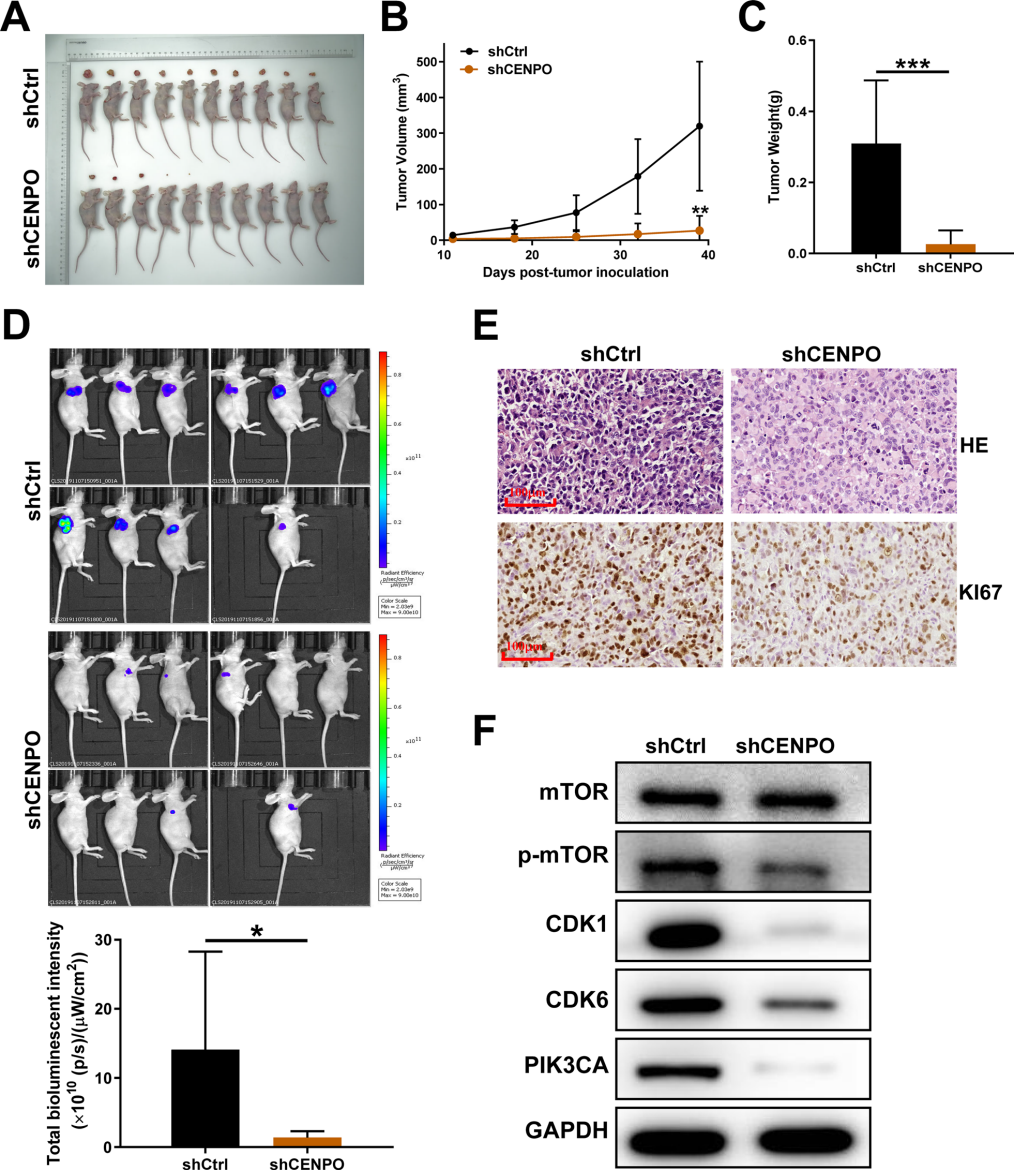
Knockdown of CENPO inhibited tumor growth in mouse xenograft models. The mouse xenograft model was established to observe the effects of CENPO knockdown on tumor volume (**A**, **B**), weight (**C**), and fluorescence expression intensity (**D**). **E** Tumor tissues were separated from mice for HE and IHC staining for detecting KI67 expression in mouse tumor tissues of shCENPO group and shCtrl group. Scale bar = 100 µm. **F** The expression of the target proteins of mTOR pathways was observed by western blotting in NCI-H1299 cells. The protein blot images are cropped. The presented results were representative of experiments repeated at least three times. Data was represented as mean ± SD. *P < 0.05, **P < 0.01, ***P < 0.001

Through continuous monitoring of mice xenograft model founding that the tumor volume of mice in the shCENPO group was remarkably smaller than that in the shCtrl group (P < 0.05) (Figs. 14A and B). After CENPO knockdown, the average weight of tumor decreased obviously (Fig. 14C). The monitoring results on day showed that the fluorescence intensity in shCENPO group was significantly higher than in shCtrl group (Fig. 14D). Subsequently, IHC and HE staining showed that the number of tumor cells expressing the KI67 signal and inflammatory injury in the shCENPO group was significantly less than that in the shCtrl group (Fig. 14E). As a consequence, these results suggested that the down-regulation of CENPO expression can lead to weak growth both in vitro and in vivo.

In addition, we briefly summarize the results of exploration on the downstream signaling pathway. The reduced expression of CENPO attenuated the mTOR phosphorylation level, negatively regulated CDK1, CDK6, and PIK3CA in NCI-H1299 cells (Fig. 14F). Therefore, we suggested that CENPO knockdown might inhibit LUAD by mediating PIK3CA /mTOR signaling pathway.

## Discussion

We found that CENPO was significantly upregulated and was correlated with the clinical stage in several cancers, and the ROC curve analysis showed that CENPO may function as a diagnostic biomarker. Prognostic analysis showed that increased CENPO was related to poorer OS in ACC, KICH, LIHC, LUAD, SARC, UVM, LGG, SKCM, and MESO. Mutation analysis showed that CENPO mutation was associated with a worse OS of BLCA. Among them, we found that CENPO was significantly up-regulated and correlated with the stage of LUAD. Thus, we chose LUAD to verify our bioinformatics result. In in vitro experiments, CENPO was significantly up-regulated in LUAD tissues. In terms of the mechanism, the GO and KEGG analyses suggest that CENPO was correlated with cell cycle and DNA replication, and the single-cell sequence data also suggest that CENPO was positively associated with cell cycle, DNA damage, DNA repair, invasion, and proliferation. The correlation analysis suggested a positive correlation between CENPO and cell cycle-related proteins, including CDK1, CDK2, CDK4, CDK6, CCNA, CCNB, CCNC, and CCNE. Meanwhile, our in vitro experiments also suggest that the knockdown of CENPO may significantly suppress the proliferation abilities of A549 and HCI-H1299 cells, and induced G2/M arrest and apoptosis, which was further confirmed using a subcutaneous xenograft tumor model in vivo. These results suggest that CENPO may be involved in the pathogenesis and pathological progression of LUAD.

The ability of CENPO to inhibit tumorigenesis has prompted us to explore downstream pathways that may mediate CENPO carcinogenesis. In this study, we demonstrated that CENPO knockdown decreased expression of downstream protein p-mTOR, CDK1, CDK6, PIK3CA (PI3K). Previous studies had shown that activation of mTOR promotes tumor growth and metastasis [17]. The PIK3CA mutation is also a common feature of LUAD, which is related to a poor prognosis [18]. Alterations in the cell cycle and PI3K pathways were associated with recurrence of LUAD [19]. The PI3K/Akt/mTOR pathway regulates cell proliferation, growth, cell size, metabolism, and motility, of which component genes have been extensively studied and found to be commonly activated in human cancer and inhibition of this pathway has been shown to lead to regression of human tumors [20]. Furthermore, p-mTOR, PIK3CA, CDK1 and CDK6 were significantly downregulated after the knockdown of CENPO. CDK1/6 are members of the CDK family and play significant roles in cell cycle and proliferation regulation [21, 22]. Cyclin E(CCNE)/CDK2 complexes appear to have several other critical roles in cell cycle progression [23]. Therefore, our studies show that CENPO may significantly suppressed the PI3K / mTOR signaling pathway, and induce G2 arrest and apoptosis of A549 and HCI-H1299 cells. CENPO may function as a potential biomarker and therapy target of LUAD.

Our results also show that CENPO plays an essential role in cancer immunity. Tumor microenvironment (TME) characteristics serve as markers for evaluating tumor cell responses to immunotherapy and influence clinical outcomes [24]. The immune cells of TME are associated with the effect of immunotherapy, including CD4 + T cells, CD8 + T cells, MDSCs, and NKT cells [25–28]. According to ESTIMATE scores, there were positive correlations between CENPO expression and immune cell content in the TME of several cancers. Tumor-infiltrating immune cells play an important impact on tumor formation and development and can antagonize or promote tumorigenesis [29]. In LUAD, the obtained results show that there was a negative association between CENPO and the infiltration levels of MDSCs, T-cell NK cells, CD8 + T cells, Tregs, B cells, Myeloid dendritic cells, Monocytes and Macrophage M2. Furthermore, the single-cell sequencing data also showed that CENPO was positively associated with cell cycle, proliferation, DNA damage, DNA repair and invasion, but was negatively correlated with the inflammation and differentiation of LUAD. Therefore, CENPO is associated with the degree of immune cell infiltration and may be a potential biomarker for LUAD immunotherapy.

However, our study still has some limitations. Our findings suggest that CENPO is involved in the infiltration of immune cells in LUAD, but the mechanism is unclear and requires further investigation.

## Conclusion

In pan-cancer, especially LUAD, CENPO may be a potential biomarker and oncogene. Furthermore, CENPO is involved in the infiltration of immune cells into pan-cancer, making it a potential immunotherapeutic target in cancer therapy. Additionally, downregulation of CENPO inhibited the malignant progression of LUAD cells, such as reduced proliferation, cycle repression in the G2 phase, increased apoptotic sensitivity, and inhibition of migration. In vivo experiments further confirmed that CENPO down-regulation attenuated tumor growth. Reduced expression of CENPO attenuated the phosphorylation level of mTOR, negatively regulated CDK1, CDK6, and PIK3CA. In summary, the main discovery was the determination of promoting role of the CENPO in LUAD, demonstrating that small molecule inhibitors targeting CENPO were a novel therapeutic strategy for LUAD.

## Supplementary Information

**Additional file 1:** Public database.

**Additional file 2: Figure S1.** Differential expression of CENPO. (A) Gene transcript exon expression of CENPO and (B) isoform expression of CENPO in the GTEx database. (C) Single-tissue eQTL of CENPO tissue specific expression. (D) Single cell expression of CENPO. **Figure S2.** The diagnostic value of CENPO in pan-cancer. (A) The expression of CENPO in the Sangerbox database. (B) The ROC curves of CENPO in ACC, BLCA, BRCA, CESC, CHOL, ESCA, GBM, HNSC, KICH, UCEC, KIRP, LGG, LIHC, LUAD, LUSC, and OV. **Figure S3.** The prognosis value of CENPO in pan-cancer. (A) Kaplan–Meier analysis of the association between CENPO expression and overall survival (OS). (B) Kaplan–Meier analysis of the association between CENPO expression and disease-free survival (DFS). **Figure S4.** The association between CENPO expression and immune cell infiltration, including T-cell NK cells, CD8 + T cells, Tregs, B cells, Myeloid dendritic cells, Monocytes and Macrophage M2**. Figure S5.** The correlation between CENPO expression and major histocompatibility complexes (MHCs) in the TISIDB database. (A) The expression of CENPO is negatively associated with most MHCs in pan-cancer. (B) The expression of CENPO is negatively associated with most chemokine receptors in LUAD. **Figure S6.** (A) The mutation annotation format (MAF) summary plots of CENPO in the CENPO^high^ group and CENPO^low^ group. (B) OS, DFS, DSS, and PFS analysis stratified by CENPO mutation status in bladder urothelial carcinoma (BLCA). **Figure S7.** CENPO is decreased in shRNA mediated knockdown of A549 and HCI-H1299 cells. The specificity and validity of the lentivirus-mediated shRNA knockdown of CENPO in A549 and HCI-H1299 cells was measured by RT-qPCR (A) and Western blot (B). The protein blot images are cropped.

**Additional file 3: Table S1**. The value of ROC, DFS, and OS curves of CENPO in multiple types of cancers.

**Additional file 4: Table S2**. The correlation of CENPO expression and ESTIMATEScore, ImmuneScore, and StromalScore in multiple types of cancers.

**Additional file 5: Table S3**. The relationship between CENPO expression and 22 immune cells in multiple types of cancers.

**Additional file 6: Table S4**. The correlation of CENPO expression with TMB, MSI, and MATH in multiple types of cancers.

**Additional file 7: Table S5**. The expressions of CENPO and 60 immune checkpoint (ICP) genes in multiple types of cancers.

**Additional file 8: Table S6**. The correlation value of CENPO with the expressions of 60 immune checkpoint (ICP) genes in multiple types of cancers.

## Data Availability

The original contributions presented in the study are included in the article/Additional file 1: Material S1, Additional files 3, 4, 5, 6, 7: Table S1–S5. Further inquiries can be directed to the corresponding authors.

## Abbreviations

CENPO: Centromere protein O
LUAD: Lung adenocarcinoma
IHC: Immunohistochemical
MDSC: Myeloid-derived suppressor cells
OS: Overall survival
TCGA: The Cancer Genome Atlas
TME: Tumor microenvironment
ICP: Immune checkpoint
CTEx: Genotype Tissue Expression
TMB: Tumor mutation burden
MSI: Microsatellite instability
MATH: Mutant-allele tumor heterogeneity
CNAs: Copy number alternations
GSVA: Gene set variation analysis
GO: Gene ontology
KEGG: Kyoto Encyclopedia of Genes and Genomes
