# Supplementary material for: Pan-cancer landscape of CENPO and its underlying mechanism in LUAD
Source: Respir Res. 2023 Apr 15;24:113. doi: 10.1186/s12931-023-02408-3 (PMC10105544; doi:10.1186/s12931-023-02408-3)
Supplement: Supplementary file 1 — Additional file 1: Public database. [file 12931_2023_2408_MOESM1_ESM.docx]

Additional file for

***Pan-cancer landscape of CENPO and its underlying mechanism in LUAD***

***Public database***

In our article, Sangerbox^1^ and GEPIA^2^ database are the main basis.

Sangerbox, a web analysis tool, integrates a variety of common tumor data, including GEO, TCGA, ICGC, and GTEx. The analysis content covers gene differential expression analysis, survival analysis, immune infiltration, immune checkpoint and co-expression network. Our study mainly applies the pan-cancer difference analysis, survival analysis, tumor heterogeneity analysis and checkpoint analysis based on this database.

GEPIA is also a common tumor web tool, which covers GEO, TCGA, ICGC, and GTEx. Its analysis content includes gene differential expression analysis, correlation analysis, patient survival analysis, similar gene detection and dimensionality reduction analysis. In this study, we mainly applied the pan-cancer difference analysis, survival analysis based on the database.

Many online databases are used for tumor feature analysis and construction of ceRNA network. See the following table for details:

| database | full name | function | source | study |
| --- | --- | --- | --- | --- |
| Sangerbox |  | The platform as a user-friendly interface supports differential analysis and provides interactive customizable analysis tools, including various kinds of correlation analyses, pathway enrichment analysis, weighted correlation network analysis as well as some other common tools and functions. Users only need. | <http://www.sangerbox.com/> | ^1^ |
| TCGA | The Cancer Genome Atlas | The GDC Data Portal is a robust data-driven platform that allows cancer researchers and bioinformaticians to search and download cancer data for analysis. | <https://portal.gdc.cancer.gov/> | ^3^ |
| GTEx | The Genotype-Tissue Expression | The Genotype-Tissue Expression (GTEx) project was established to characterize genetic effects on the transcriptome across human tissues and to link these regulatory mechanisms to trait and disease associations. | <https://gtexportal.org/home/> | ^4^ |
| GEPIA | Gene Expression Profiling Interactive Analysis | GEPIA is a newly developed interactive web server for analyzing the RNA sequencing expression data of 9,736 tumors and 8,587 normal samples from the TCGA and the GTEx projects, using a standard processing pipeline. | <http://gepia.cancer-pku.cn/> | ^2^ |
| cBioPortal |  | The cBioPortal software is now available under an open-source license via GitHub. The software is now developed and maintained by a multi-institutional team, consisting of MSK, the Dana Farber Cancer Institute, Princess Margaret Cancer Centre in Toronto, Children's Hospital of Philadelphia, Caris Life Sciences, The Hyve and SE4BIO in the Netherlands, and Bilkent University in Ankara, Turkey. | <https://docs.cbioportal.org/about-us/> | ^5^ |
| LinkedOmics |  | LinkedOmics is publicly available portal that includes multi-omics data from all 32 TCGA Cancer types. It also includes mass spectrometry-based proteomics data generated by the Clinical Proteomics Tumor Analysis Consortium (CPTAC) for TCGA breast, colorectal and ovarian tumors. | [http://www.linkedomics.org](http://www.linkedomics.org/) | ^6^ |
| CancerSEA |  | CancerSEA is the first dedicated database that aims to comprehensively decode distinct functional states of cancer cells at single-cell resolution. | <http://biocc.hrbmu.edu.cn/CancerSEA/> | ^7^ |
| GSEA | Gene Set Enrichment Analysis | Gene Set Enrichment Analysis (GSEA) is a computational method that determines whether an a priori defined set of genes shows statistically significant, concordant differences between two biological states (e.g. phenotypes). | <http://www.gsea-msigdb.org/gsea/msigdb/index.jsp> | ^8^ |
| TISIDB |  | TISIDB is a web portal for tumor and immune system interaction, which integrates multiple heterogeneous data types. | <http://cis.hku.hk/TISIDB/index.php> | ^9^ |
| miRWalk |  | miRWalk stores predicted data obtained with a machine learning algorithm including experimentally verified miRNA-target interactions. The focus lies on accuracy, simplicity, user-friendly design and mostly up to date informations. | <http://mirwalk.umm.uni-heidelberg.de/> | ^10^ |
| miRDB | microRNA Target Prediction Database | miRDB is an online database for miRNA target prediction and functional annotations. | <https://mirdb.org/> | ^11^ |
| TargetScan |  | Search for predicted microRNA targets in mammals | <https://www.targetscan.org/vert_71/> | ^12^ |
| DIANA-LncBase |  | DIANA-LncBase v3 is a reference repository with experimentally supported miRNA targets on non-coding transcripts. It catalogues approximately ~500,000 entries, corresponding to ~240,000 unique tissue and cell-type specific miRNA-lncRNA interactions. | <https://diana.e-ce.uth.gr/lncbasev3> | ^13^ |

***CENPO and lung adenocarcinoma***

**Step1. CENPO may be involved in tumor progression and prognosis**

1. The expression of CENPO in pan-cancer

The expression of CENPO in different tissues is from GTEx database, and the gene differential expression analysis between tumor and normal group is based on GEPIA database. The pan-cancer expression of CENPO in different levels is based on GEPIA database. The results showed that the expression of CENPO in lung adenocarcinoma was closely related to the tumor malignancy.

1. Prognosis of CENPO in pan-cancer

The prognosis ROC of CENPO in pan-cancer is based on the R package *pROC^14^*. ROC>0.5 is represent that CENPO is the important joiner for tumor prognosis. Overall survival (OS) and disease-free survival (DFS) data are from GEPIA database.

**Step2. CENPO and tumor immune mechanism**

1. CENPO and immune cells

In pan-cancer, the relationship between the CENPO expression value and immune cell fraction comes from the SangerBox database, where *p*<0.05 shows that the CENPO expression is related to this immune cell.

1. CENPO and immune estimates

The relationship between the CENPO expression value and immune estimates is based on the SangerBox database.

1. CENPO and immune checkpoint

The relationship between the CENPO expression value and immune checkpoint is based on the SangerBox database.

1. CENPO and chemokines and chemokine receptors

The relationship between the CENPO expression value and chemokines and chemokine receptors comes from TISIDB database.

**Step3. CENPO and tumor heterogeneity**

The heterogeneity index of pan-cancer (TMB, MSI and MATH) is from the SangerBox database, and the red ones represent the significantly correlated between tumor heterogeneity and CENPO.

**Step4. CENPO and lung adenocarcinoma**

4.1 Classification of lung adenocarcinoma based on CENPO expression

According to the CENPO expression in different lung cancer patients, we divided lung adenocarcinoma into two subtypes: high expression of CENPO and low expression of CENPO.

4.2 Mutation landscape of CENPO high and low subtypes in lung adenocarcinoma

The mutation data of CENPO in pan-cancer is based on the cBioPortal tool online database. The mutation landscape analysis of CENPO high and low subtypes is based on R package *maftools^15^*.

4.3 Epigenetic mechanism of CENPO in lung adenocarcinoma

According to miRWalk (http://mirwalk.umm.uni-heidelberg.de/), miRDB (http://mirdb.org/), and TargetScan (<http://www.targetscan.org/vert_72/>). The upstream regulatory miRNA of CENPO was determined, and the lncRNA interacting with miRNA was determined according to the LncBase database to construct the apparent regulatory network of CENPO.

**Reference**

1 Shen, W. *et al.* Sangerbox: A comprehensive, interaction-friendly clinical bioinformatics analysis platform. *iMeta* **1**, e36, doi:<https://doi.org/10.1002/imt2.36> (2022).

2 Li, C., Tang, Z., Zhang, W., Ye, Z. & Liu, F. GEPIA2021: integrating multiple deconvolution-based analysis into GEPIA. *Nucleic Acids Res* **49**, W242-w246, doi:10.1093/nar/gkab418 (2021).

3 Liu, J. *et al.* An Integrated TCGA Pan-Cancer Clinical Data Resource to Drive High-Quality Survival Outcome Analytics. *Cell* **173**, 400-416.e411, doi:10.1016/j.cell.2018.02.052 (2018).

4 de Goede, O. M. *et al.* Population-scale tissue transcriptomics maps long non-coding RNAs to complex disease. *Cell* **184**, 2633-2648.e2619, doi:10.1016/j.cell.2021.03.050 (2021).

5 Wu, P. *et al.* Integration and Analysis of CPTAC Proteomics Data in the Context of Cancer Genomics in the cBioPortal. *Mol Cell Proteomics* **18**, 1893-1898, doi:10.1074/mcp.TIR119.001673 (2019).

6 Boroń, D. *et al.* Recent Multiomics Approaches in Endometrial Cancer. *Int J Mol Sci* **23**, doi:10.3390/ijms23031237 (2022).

7 Yuan, H. *et al.* CancerSEA: a cancer single-cell state atlas. *Nucleic Acids Res* **47**, D900-d908, doi:10.1093/nar/gky939 (2019).

8 Reimand, J. *et al.* Pathway enrichment analysis and visualization of omics data using g:Profiler, GSEA, Cytoscape and EnrichmentMap. *Nat Protoc* **14**, 482-517, doi:10.1038/s41596-018-0103-9 (2019).

9 Ru, B. *et al.* TISIDB: an integrated repository portal for tumor-immune system interactions. *Bioinformatics* **35**, 4200-4202, doi:10.1093/bioinformatics/btz210 (2019).

10 Sticht, C., De La Torre, C., Parveen, A. & Gretz, N. miRWalk: An online resource for prediction of microRNA binding sites. *PLoS One* **13**, e0206239, doi:10.1371/journal.pone.0206239 (2018).

11 Chen, Y. & Wang, X. miRDB: an online database for prediction of functional microRNA targets. *Nucleic Acids Res* **48**, D127-d131, doi:10.1093/nar/gkz757 (2020).

12 Rawat, M. *et al.* IL1B Increases Intestinal Tight Junction Permeability by Up-regulation of MIR200C-3p, Which Degrades Occludin mRNA. *Gastroenterology* **159**, 1375-1389, doi:10.1053/j.gastro.2020.06.038 (2020).

13 Paraskevopoulou, M. D. *et al.* DIANA-LncBase v2: indexing microRNA targets on non-coding transcripts. *Nucleic Acids Res* **44**, D231-238, doi:10.1093/nar/gkv1270 (2016).

14 Jiang, J. *et al.* Hsa_circ_0015278 Regulates FLT3-ITD AML Progression via Ferroptosis-Related Genes. *Cancers (Basel)* **15**, doi:10.3390/cancers15010071 (2022).

15 Mayakonda, A., Lin, D. C., Assenov, Y., Plass, C. & Koeffler, H. P. Maftools: efficient and comprehensive analysis of somatic variants in cancer. *Genome Res* **28**, 1747-1756, doi:10.1101/gr.239244.118 (2018).
