# Supplementary figures and images for: Pan-cancer landscape of CENPO and its underlying mechanism in LUAD
Source: Respir Res. 2023 Apr 15;24:113. doi: 10.1186/s12931-023-02408-3 (PMC10105544; doi:10.1186/s12931-023-02408-3)

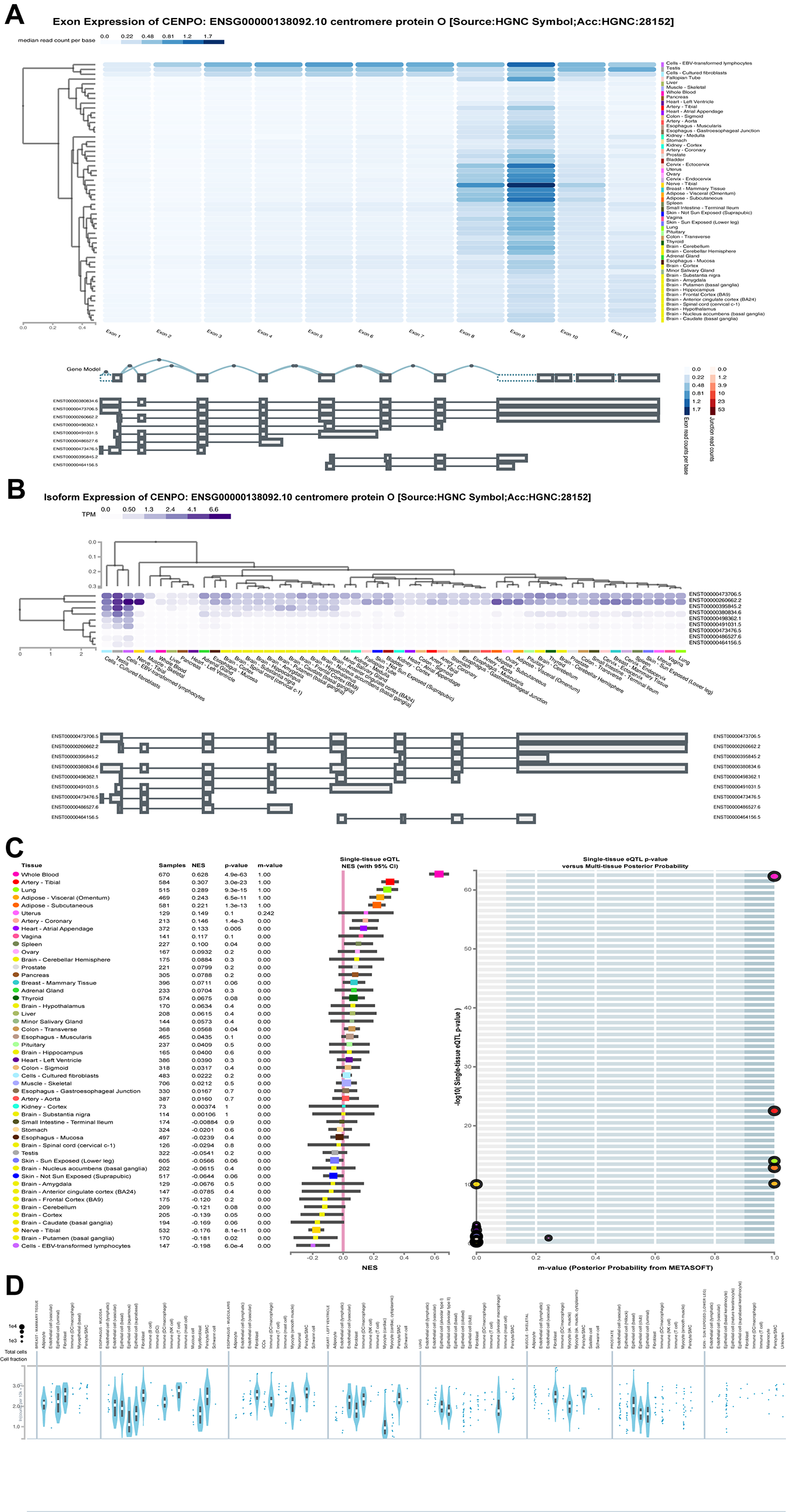

Supplement: Supplementary file 2 — Additional file 2: Figure S1. Differential expression of CENPO. (A) Gene transcript exon expression of CENPO and (B) isoform expression of CENPO in the GTEx database. (C) Single-tissue eQTL of CENPO tissue specific expression. (D) Single cell expression of CENPO. Figure S2. The diagnostic value of CENPO in pan-cancer. (A) The expression of CENPO in the Sangerbox database. (B) The ROC curves of CENPO in ACC, BLCA, BRCA, CESC, CHOL, ESCA, GBM, HNSC, KICH, UCEC, KIRP, LGG, LIHC, LUAD, LUSC, and OV. Figure S3. The prognosis value of CENPO in pan-cancer. (A) Kaplan–Meier analysis of the association between CENPO expression and overall survival (OS). (B) Kaplan–Meier analysis of the association between CENPO expression and disease-free survival (DFS). Figure S4. The association between CENPO expression and immune cell infiltration, including T-cell NK cells, CD8 + T cells, Tregs, B cells, Myeloid dendritic cells, Monocytes and Macrophage M2. Figure S5. The correlation between CENPO expression and major histocompatibility complexes (MHCs) in the TISIDB database. (A) The expression of CENPO is negatively associated with most MHCs in pan-cancer. (B) The expression of CENPO is negatively associated with most chemokine receptors in LUAD. Figure S6. (A) The mutation annotation format (MAF) summary plots of CENPO in the CENPOhigh group and CENPOlow group. (B) OS, DFS, DSS, and PFS analysis stratified by CENPO mutation status in bladder urothelial carcinoma (BLCA). Figure S7. CENPO is decreased in shRNA mediated knockdown of A549 and HCI-H1299 cells. The specificity and validity of the lentivirus-mediated shRNA knockdown of CENPO in A549 and HCI-H1299 cells was measured by RT-qPCR (A) and Western blot (B). The protein blot images are cropped. [file 12931_2023_2408_MOESM2_ESM.zip › Supplementary Figures/Figure S1.tif]

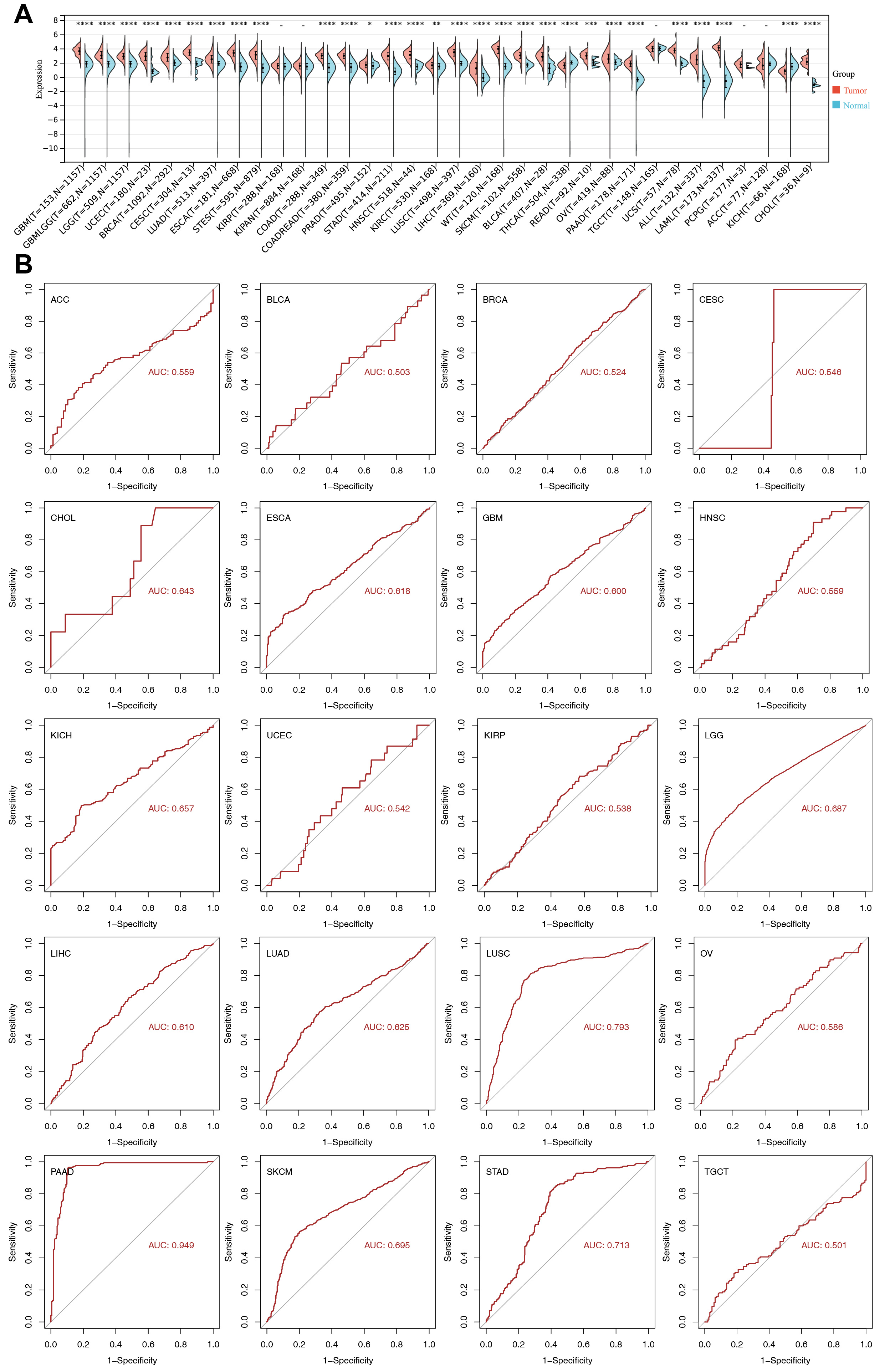

Supplement: Supplementary file 2 — Additional file 2: Figure S1. Differential expression of CENPO. (A) Gene transcript exon expression of CENPO and (B) isoform expression of CENPO in the GTEx database. (C) Single-tissue eQTL of CENPO tissue specific expression. (D) Single cell expression of CENPO. Figure S2. The diagnostic value of CENPO in pan-cancer. (A) The expression of CENPO in the Sangerbox database. (B) The ROC curves of CENPO in ACC, BLCA, BRCA, CESC, CHOL, ESCA, GBM, HNSC, KICH, UCEC, KIRP, LGG, LIHC, LUAD, LUSC, and OV. Figure S3. The prognosis value of CENPO in pan-cancer. (A) Kaplan–Meier analysis of the association between CENPO expression and overall survival (OS). (B) Kaplan–Meier analysis of the association between CENPO expression and disease-free survival (DFS). Figure S4. The association between CENPO expression and immune cell infiltration, including T-cell NK cells, CD8 + T cells, Tregs, B cells, Myeloid dendritic cells, Monocytes and Macrophage M2. Figure S5. The correlation between CENPO expression and major histocompatibility complexes (MHCs) in the TISIDB database. (A) The expression of CENPO is negatively associated with most MHCs in pan-cancer. (B) The expression of CENPO is negatively associated with most chemokine receptors in LUAD. Figure S6. (A) The mutation annotation format (MAF) summary plots of CENPO in the CENPOhigh group and CENPOlow group. (B) OS, DFS, DSS, and PFS analysis stratified by CENPO mutation status in bladder urothelial carcinoma (BLCA). Figure S7. CENPO is decreased in shRNA mediated knockdown of A549 and HCI-H1299 cells. The specificity and validity of the lentivirus-mediated shRNA knockdown of CENPO in A549 and HCI-H1299 cells was measured by RT-qPCR (A) and Western blot (B). The protein blot images are cropped. [file 12931_2023_2408_MOESM2_ESM.zip › Supplementary Figures/Figure S2.jpg]

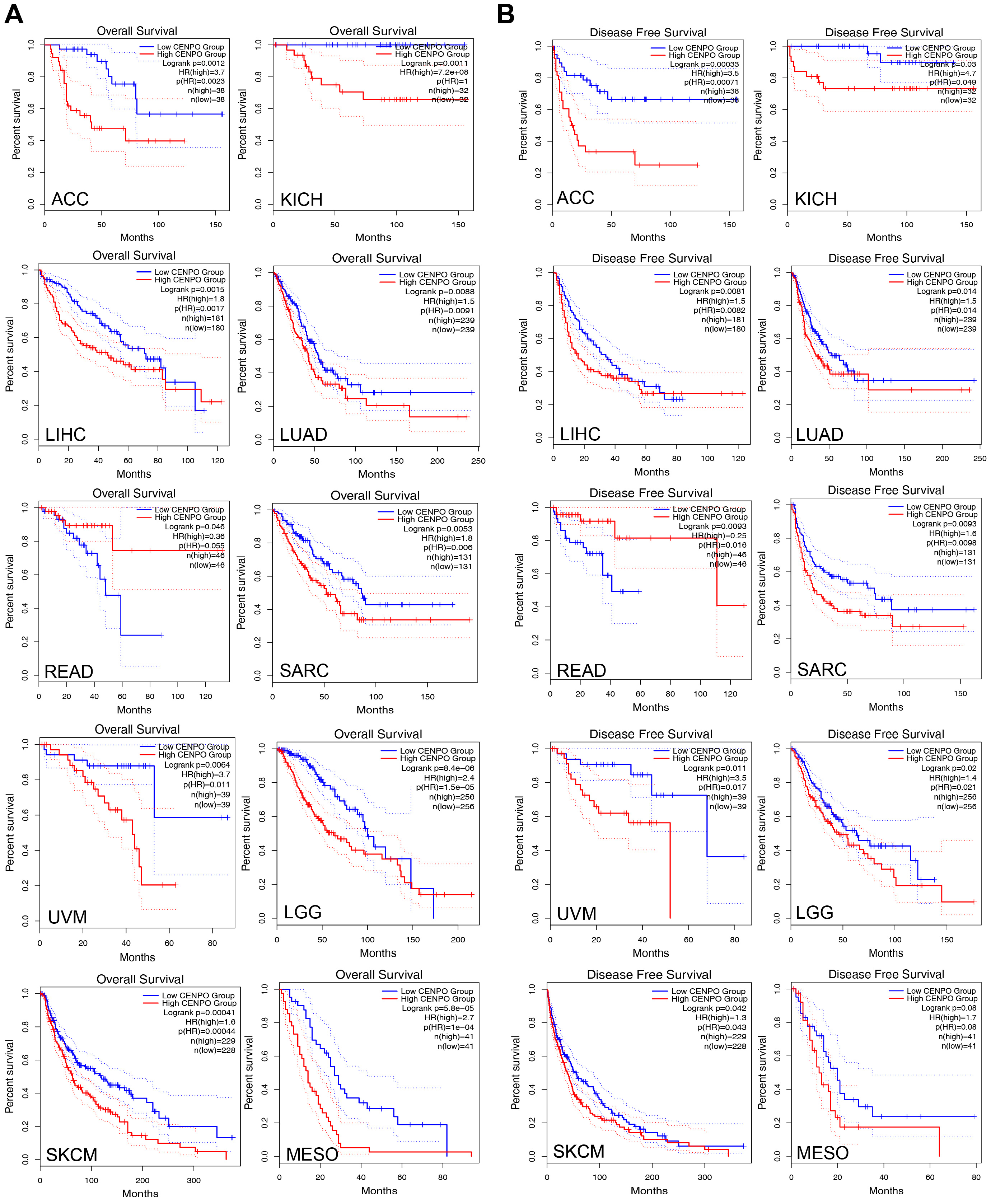

Supplement: Supplementary file 2 — Additional file 2: Figure S1. Differential expression of CENPO. (A) Gene transcript exon expression of CENPO and (B) isoform expression of CENPO in the GTEx database. (C) Single-tissue eQTL of CENPO tissue specific expression. (D) Single cell expression of CENPO. Figure S2. The diagnostic value of CENPO in pan-cancer. (A) The expression of CENPO in the Sangerbox database. (B) The ROC curves of CENPO in ACC, BLCA, BRCA, CESC, CHOL, ESCA, GBM, HNSC, KICH, UCEC, KIRP, LGG, LIHC, LUAD, LUSC, and OV. Figure S3. The prognosis value of CENPO in pan-cancer. (A) Kaplan–Meier analysis of the association between CENPO expression and overall survival (OS). (B) Kaplan–Meier analysis of the association between CENPO expression and disease-free survival (DFS). Figure S4. The association between CENPO expression and immune cell infiltration, including T-cell NK cells, CD8 + T cells, Tregs, B cells, Myeloid dendritic cells, Monocytes and Macrophage M2. Figure S5. The correlation between CENPO expression and major histocompatibility complexes (MHCs) in the TISIDB database. (A) The expression of CENPO is negatively associated with most MHCs in pan-cancer. (B) The expression of CENPO is negatively associated with most chemokine receptors in LUAD. Figure S6. (A) The mutation annotation format (MAF) summary plots of CENPO in the CENPOhigh group and CENPOlow group. (B) OS, DFS, DSS, and PFS analysis stratified by CENPO mutation status in bladder urothelial carcinoma (BLCA). Figure S7. CENPO is decreased in shRNA mediated knockdown of A549 and HCI-H1299 cells. The specificity and validity of the lentivirus-mediated shRNA knockdown of CENPO in A549 and HCI-H1299 cells was measured by RT-qPCR (A) and Western blot (B). The protein blot images are cropped. [file 12931_2023_2408_MOESM2_ESM.zip › Supplementary Figures/Figure S3.jpg]

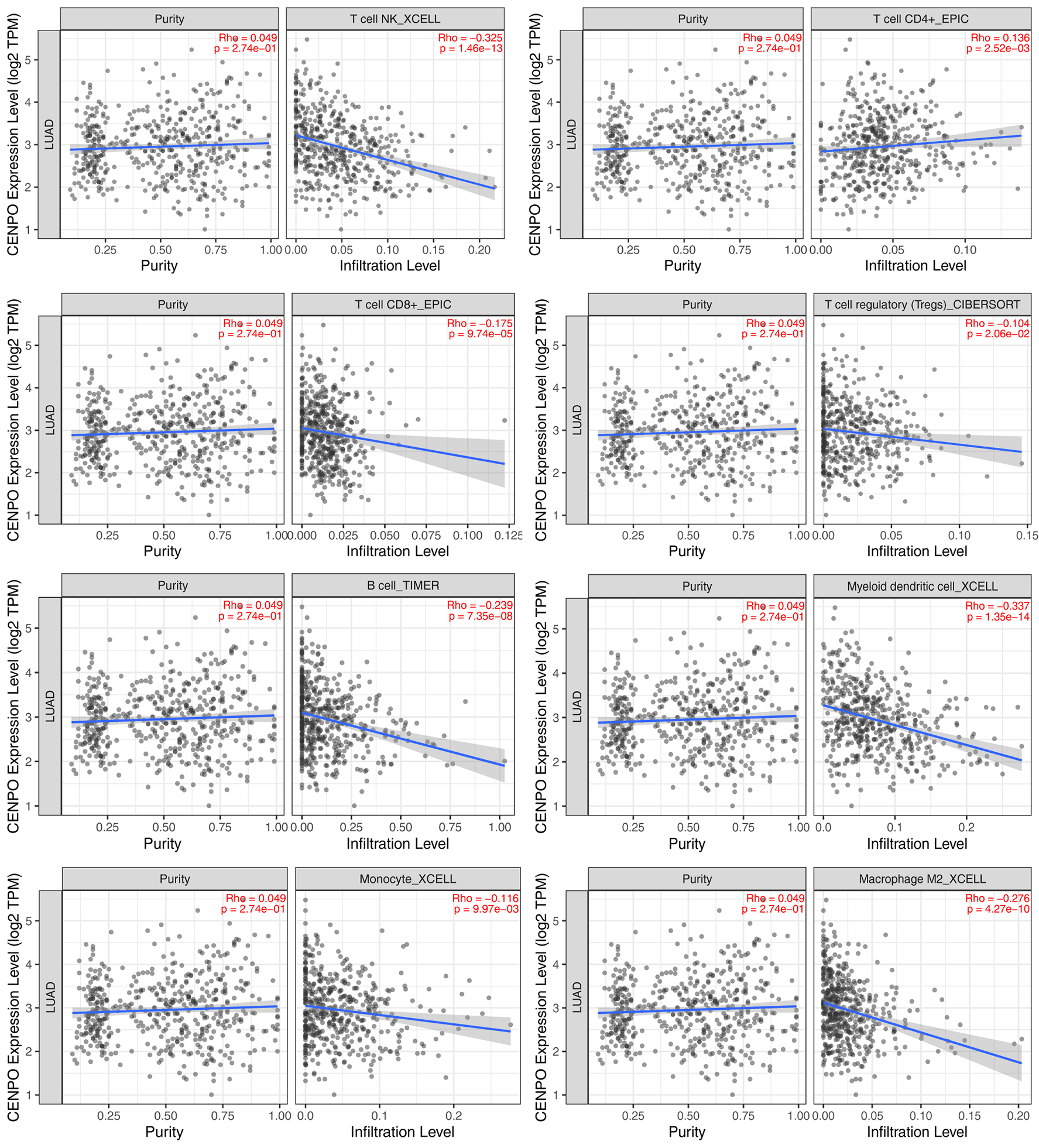

Supplement: Supplementary file 2 — Additional file 2: Figure S1. Differential expression of CENPO. (A) Gene transcript exon expression of CENPO and (B) isoform expression of CENPO in the GTEx database. (C) Single-tissue eQTL of CENPO tissue specific expression. (D) Single cell expression of CENPO. Figure S2. The diagnostic value of CENPO in pan-cancer. (A) The expression of CENPO in the Sangerbox database. (B) The ROC curves of CENPO in ACC, BLCA, BRCA, CESC, CHOL, ESCA, GBM, HNSC, KICH, UCEC, KIRP, LGG, LIHC, LUAD, LUSC, and OV. Figure S3. The prognosis value of CENPO in pan-cancer. (A) Kaplan–Meier analysis of the association between CENPO expression and overall survival (OS). (B) Kaplan–Meier analysis of the association between CENPO expression and disease-free survival (DFS). Figure S4. The association between CENPO expression and immune cell infiltration, including T-cell NK cells, CD8 + T cells, Tregs, B cells, Myeloid dendritic cells, Monocytes and Macrophage M2. Figure S5. The correlation between CENPO expression and major histocompatibility complexes (MHCs) in the TISIDB database. (A) The expression of CENPO is negatively associated with most MHCs in pan-cancer. (B) The expression of CENPO is negatively associated with most chemokine receptors in LUAD. Figure S6. (A) The mutation annotation format (MAF) summary plots of CENPO in the CENPOhigh group and CENPOlow group. (B) OS, DFS, DSS, and PFS analysis stratified by CENPO mutation status in bladder urothelial carcinoma (BLCA). Figure S7. CENPO is decreased in shRNA mediated knockdown of A549 and HCI-H1299 cells. The specificity and validity of the lentivirus-mediated shRNA knockdown of CENPO in A549 and HCI-H1299 cells was measured by RT-qPCR (A) and Western blot (B). The protein blot images are cropped. [file 12931_2023_2408_MOESM2_ESM.zip › Supplementary Figures/Figure S4.tif]

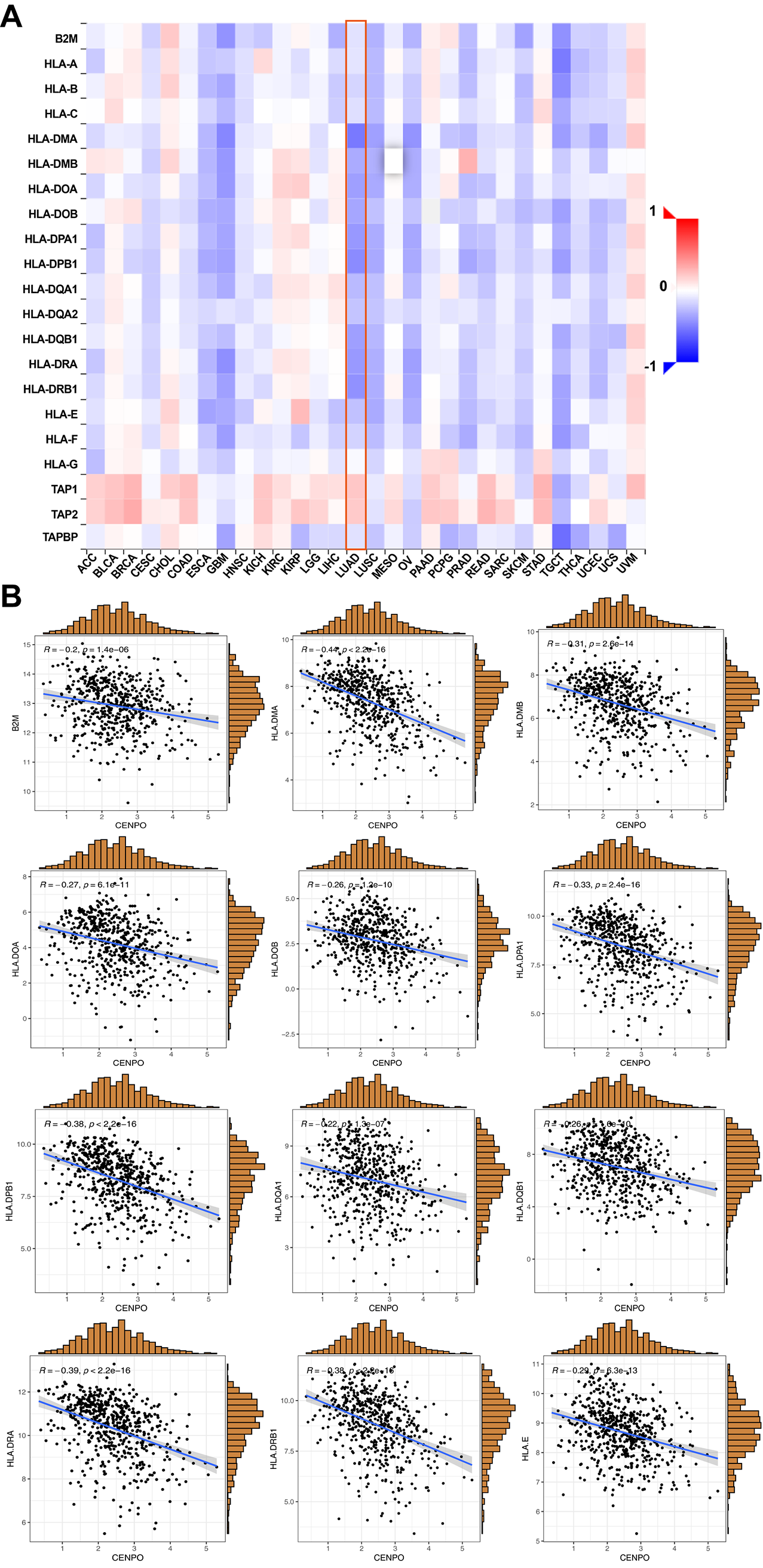

Supplement: Supplementary file 2 — Additional file 2: Figure S1. Differential expression of CENPO. (A) Gene transcript exon expression of CENPO and (B) isoform expression of CENPO in the GTEx database. (C) Single-tissue eQTL of CENPO tissue specific expression. (D) Single cell expression of CENPO. Figure S2. The diagnostic value of CENPO in pan-cancer. (A) The expression of CENPO in the Sangerbox database. (B) The ROC curves of CENPO in ACC, BLCA, BRCA, CESC, CHOL, ESCA, GBM, HNSC, KICH, UCEC, KIRP, LGG, LIHC, LUAD, LUSC, and OV. Figure S3. The prognosis value of CENPO in pan-cancer. (A) Kaplan–Meier analysis of the association between CENPO expression and overall survival (OS). (B) Kaplan–Meier analysis of the association between CENPO expression and disease-free survival (DFS). Figure S4. The association between CENPO expression and immune cell infiltration, including T-cell NK cells, CD8 + T cells, Tregs, B cells, Myeloid dendritic cells, Monocytes and Macrophage M2. Figure S5. The correlation between CENPO expression and major histocompatibility complexes (MHCs) in the TISIDB database. (A) The expression of CENPO is negatively associated with most MHCs in pan-cancer. (B) The expression of CENPO is negatively associated with most chemokine receptors in LUAD. Figure S6. (A) The mutation annotation format (MAF) summary plots of CENPO in the CENPOhigh group and CENPOlow group. (B) OS, DFS, DSS, and PFS analysis stratified by CENPO mutation status in bladder urothelial carcinoma (BLCA). Figure S7. CENPO is decreased in shRNA mediated knockdown of A549 and HCI-H1299 cells. The specificity and validity of the lentivirus-mediated shRNA knockdown of CENPO in A549 and HCI-H1299 cells was measured by RT-qPCR (A) and Western blot (B). The protein blot images are cropped. [file 12931_2023_2408_MOESM2_ESM.zip › Supplementary Figures/Figure S5.tif]

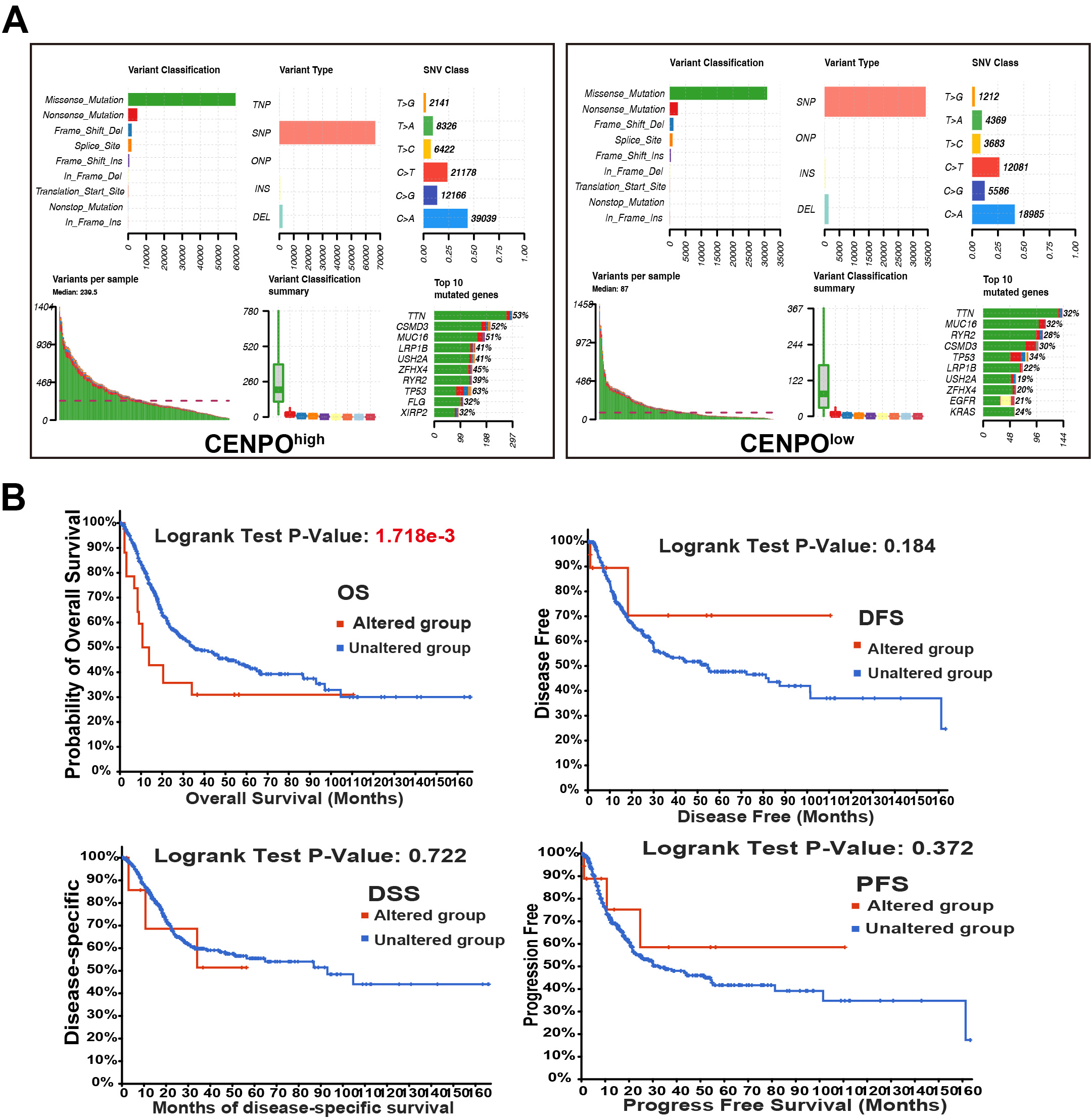

Supplement: Supplementary file 2 — Additional file 2: Figure S1. Differential expression of CENPO. (A) Gene transcript exon expression of CENPO and (B) isoform expression of CENPO in the GTEx database. (C) Single-tissue eQTL of CENPO tissue specific expression. (D) Single cell expression of CENPO. Figure S2. The diagnostic value of CENPO in pan-cancer. (A) The expression of CENPO in the Sangerbox database. (B) The ROC curves of CENPO in ACC, BLCA, BRCA, CESC, CHOL, ESCA, GBM, HNSC, KICH, UCEC, KIRP, LGG, LIHC, LUAD, LUSC, and OV. Figure S3. The prognosis value of CENPO in pan-cancer. (A) Kaplan–Meier analysis of the association between CENPO expression and overall survival (OS). (B) Kaplan–Meier analysis of the association between CENPO expression and disease-free survival (DFS). Figure S4. The association between CENPO expression and immune cell infiltration, including T-cell NK cells, CD8 + T cells, Tregs, B cells, Myeloid dendritic cells, Monocytes and Macrophage M2. Figure S5. The correlation between CENPO expression and major histocompatibility complexes (MHCs) in the TISIDB database. (A) The expression of CENPO is negatively associated with most MHCs in pan-cancer. (B) The expression of CENPO is negatively associated with most chemokine receptors in LUAD. Figure S6. (A) The mutation annotation format (MAF) summary plots of CENPO in the CENPOhigh group and CENPOlow group. (B) OS, DFS, DSS, and PFS analysis stratified by CENPO mutation status in bladder urothelial carcinoma (BLCA). Figure S7. CENPO is decreased in shRNA mediated knockdown of A549 and HCI-H1299 cells. The specificity and validity of the lentivirus-mediated shRNA knockdown of CENPO in A549 and HCI-H1299 cells was measured by RT-qPCR (A) and Western blot (B). The protein blot images are cropped. [file 12931_2023_2408_MOESM2_ESM.zip › Supplementary Figures/Figure S6.jpg]

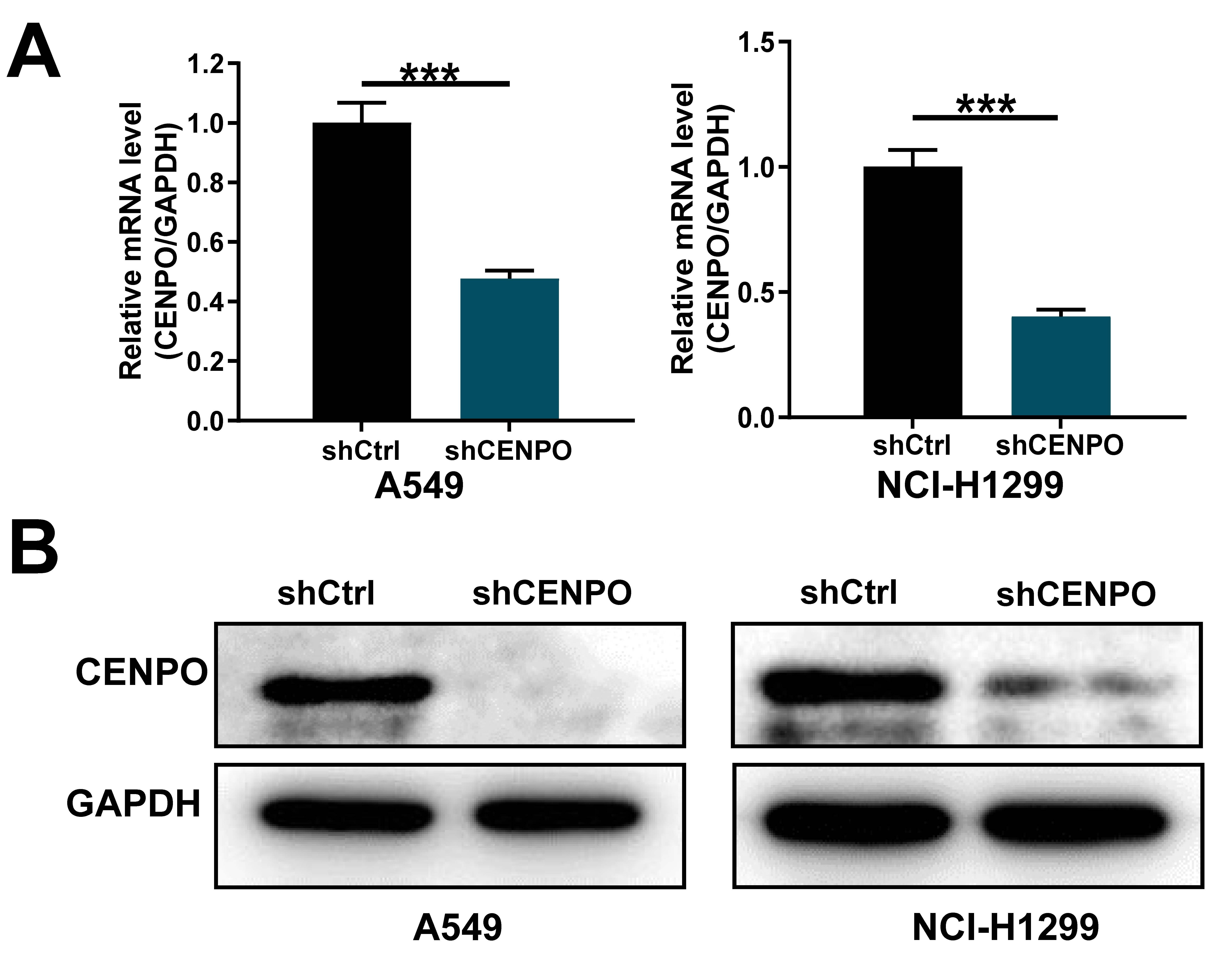

Supplement: Supplementary file 2 — Additional file 2: Figure S1. Differential expression of CENPO. (A) Gene transcript exon expression of CENPO and (B) isoform expression of CENPO in the GTEx database. (C) Single-tissue eQTL of CENPO tissue specific expression. (D) Single cell expression of CENPO. Figure S2. The diagnostic value of CENPO in pan-cancer. (A) The expression of CENPO in the Sangerbox database. (B) The ROC curves of CENPO in ACC, BLCA, BRCA, CESC, CHOL, ESCA, GBM, HNSC, KICH, UCEC, KIRP, LGG, LIHC, LUAD, LUSC, and OV. Figure S3. The prognosis value of CENPO in pan-cancer. (A) Kaplan–Meier analysis of the association between CENPO expression and overall survival (OS). (B) Kaplan–Meier analysis of the association between CENPO expression and disease-free survival (DFS). Figure S4. The association between CENPO expression and immune cell infiltration, including T-cell NK cells, CD8 + T cells, Tregs, B cells, Myeloid dendritic cells, Monocytes and Macrophage M2. Figure S5. The correlation between CENPO expression and major histocompatibility complexes (MHCs) in the TISIDB database. (A) The expression of CENPO is negatively associated with most MHCs in pan-cancer. (B) The expression of CENPO is negatively associated with most chemokine receptors in LUAD. Figure S6. (A) The mutation annotation format (MAF) summary plots of CENPO in the CENPOhigh group and CENPOlow group. (B) OS, DFS, DSS, and PFS analysis stratified by CENPO mutation status in bladder urothelial carcinoma (BLCA). Figure S7. CENPO is decreased in shRNA mediated knockdown of A549 and HCI-H1299 cells. The specificity and validity of the lentivirus-mediated shRNA knockdown of CENPO in A549 and HCI-H1299 cells was measured by RT-qPCR (A) and Western blot (B). The protein blot images are cropped. [file 12931_2023_2408_MOESM2_ESM.zip › Supplementary Figures/Figure S7.jpg]
